# Developmental and environmental determinants of hair steroids in children from birth to two years postnatally: A comprehensive analysis

**DOI:** 10.1016/j.cpnec.2026.100354

**Published:** 2026-05-27

**Authors:** Esther J. Hutloff, Isabel Jaramillo, Luisa Bergunde, Susan Garthus-Niegel, Susann Steudte-Schmiedgen

**Affiliations:** a Department of Psychotherapy and Psychosomatic Medicine, Faculty of Medicine and University Hospital Carl Gustav Carus, TUD Dresden University of Technology, Dresden, Germany; b Institute and Policlinic of Occupational and Social Medicine, Faculty of Medicine and University Hospital Carl Gustav Carus, TUD Dresden University of Technology, Dresden, Germany; c Institute for Systems Medicine (ISM), Faculty of Medicine, Medical School Hamburg MSH, Hamburg, Germany; d Department of Childhood and Families, Norwegian Institute of Public Health, Oslo, Norway

**Keywords:** Children, Hair, Steroids, Glucocorticoids, Sex hormones, Cortisol

## Abstract

**Background:**

Hair analysis is an important psychoneuroendocrine method for assessing long-term hormonal activity, yet determinants of hair steroid concentrations in early infancy remain understudied. This study evaluated the influence of developmental (sociodemographic and birth-related) as well as methodological and contextual (hair-related, organizational, and COVID-19 exposure) factors on hair steroids during the first two years of life.

**Methods:**

Children's hair samples from the prospective DREAM_HAIR_ study were collected at three weeks (*n* = 210), eight weeks (*n* = 240), 14 months (*n* = 245), and two years (*n* = 219). Cortisol (HairF), cortisone (HairE), dehydroepiandrosterone (HairDHEA), progesterone (HairP), testosterone (HairT), and corticosterone (HairB) were quantified in the proximal 2 cm hair segment using liquid chromatography–tandem mass spectrometry.

**Results:**

HairF, HairE, and HairP peaked postnatally, declined sharply within eight weeks, and decreased gradually thereafter. HairF was strongly correlated with HairE (T3–T4), while HairP and HairDHEA showed moderate to strong correlations with both HairF and HairE (T2–T4). Regression analyses revealed associations with sex (HairDHEA), hair color (HairP), season (HairE, HairDHEA), storage time (HairDHEA), gestational week (HairF), and birth mode (HairF). After correction for multiple testing, only birth mode (HairF, T1) and season (HairDHEA, T4) remained significant. Body weight and COVID-19 exposure were not related to hair steroids. High non-detectable rates for HairT and HairB limited analyses to descriptive statistics.

**Conclusion:**

Developmental, methodological and contextual influences on hair steroids were small, with consistent effects of birth mode, season, and age-related changes. Hair analysis appears to be a promising method for assessing long-term endocrine activity in early childhood.

## Highlights

• Hair cortisol, cortisone and progesterone declined with age in children aged 0-2 years.
• Sex (DHEA), gestation, and birth mode (cortisol) associated.
• Hair color (progesterone), season (cortisone, DHEA), storage time (DHEA) associated.
• Effects were small; few survived multiple testing correction.
• Hair steroid analysis is feasible in early-life endocrine assessment.

## 1 Introduction

Early developmental stages including the prenatal period and the first two years postpartum represent a critical period of neurobiological and endocrine development. During this time, the child's hypothalamic-pituitary-adrenal (HPA) and hypothalamic-pituitary-gonadal (HPG) axes mature rapidly including the secretion of steroid hormones, thus shaping stress regulation, neurodevelopment, and long-term physical and mental health [1]. Ongoing biological maturation renders these early stages especially sensitive windows, characterized by heightened susceptibility to external stressors [2]. Hormonal processes in this sensitive window including the secretion of glucocorticoids and sex steroids are essential for regulating numerous physiological processes in the human body. Glucocorticoids such as cortisol, its inactive metabolite cortisone, and corticosterone, are end products of the HPA axis and play important roles in fetal growth, development and modulation of the immune system [3] and stress adaptation [4]. Acute stress and circadian rhythm activate the HPA axis, resulting in the release of its primary end product, glucocorticoids. In the context of chronic stress, prolonged HPA axis activation may result in both hyper- and hypocortisolism [5] and impair neurodevelopment and physiological systems with lasting effects on mental and physical health. Sex steroids, including progesterone and testosterone, are regulated by the HPG axis. Together with dehydroepiandrosterone (DHEA), an adrenal steroid and a precursor of androgens and estrogens, they contribute to neurological development and neuroprotective processes [6], serve as precursors for fetal steroidogenesis [7], and are crucial for the development of secondary sex characteristics [8].

Glucocorticoids have gained considerable attention as biomarkers reflecting long-term physiological responses to sustained HPA axis activity [9]. Traditional methods measuring steroids in saliva, blood, or urine [10] are well-suited for reflecting dynamic aspects of steroid secretory activity (e.g., diurnal fluctuations, pulsatile secretion [11], and acute stress responses [12]), however provide relatively unreliable estimates of long-term steroid output. Here, the assessment of steroids in hair, pioneered by Prof. Clemens Kirschbaum and his lab among the early investigators in this field [13,14], allows for a retrospective assessment of cumulated steroids up to several months [5]. Hair sampling is feasible, painless and easily stored [15], making it particularly suitable for use in children [16]. Since young children cannot reliably report subjective stress experiences, biological measures such as hair steroid analysis appear especially valuable in this context. Thus, to strengthen reliability and interpretability of findings, it is important to better understand which developmental as well as contextual and methodological variables are relevant in determining children's hair steroid levels in this sensitive phase.

Several studies have identified factors influencing hair steroid levels in adult samples, such as age, sex, hair washing frequency, and season [2,17]. However, studies on children's hair steroids remain limited and inconsistent. Some sociodemographic variables have been identified as influential in children's hair glucocorticoid levels. For example, sex differences have been demonstrated, with boys showing higher levels of hair cortisol (HairF) [18], hair cortisone (HairE) [19], or both HairF and HairE [9]. Furthermore, studies on the link between age and HairF in children have yielded mixed findings: some found a positive association [20], others a negative one [21], or higher levels in very young children [22]. A meta-analysis by Stalder et al. [5] revealed a positive association between HairF and age, primarily spanning adolescence to adulthood, and highlighted the need for research with finer age distinctions.

Evidence on hair-related factors—such as hair washing frequency and natural hair color— in child samples is also mixed with some studies reporting lower HairF and HairE with more frequent hair washing [9], or no association [20]. Similarly, higher HairF and HairE have been found in darker hair [9], though other studies found no effect of natural hair color [20]. In adults, reductions in HairF and HairE have been observed with sunlight exposure [11,23]. Additionally, seasonal variations in HairF, with higher levels in summer and fall [24] or summer and spring [17] compared to winter, have been reported. In children, however, the effects of sunlight exposure and season on hair steroids remain understudied. Preliminary findings suggest a negative association between sun exposure and HairE in 6-year-old children [9].

Besides hair-related factors, studies on adult hair samples have considered the potential influence of organizational effects, such as storage time—the interval between hair collection and laboratory analysis—reporting a negative association with HairF [[25], [26], [27]] and HairE [28,29].

Considering the early maturation of the HPA axis, preliminary studies have indicated that birth-related variables (i.e., gestational age, birth weight, birth mode) may be associated with individual variability in hair glucocorticoids beyond the neonatal period. Some studies, including samples of infants aged 0**–**7 weeks, demonstrated that gestational age and higher birth weight are associated with higher HairF and HairE [30], while others reported no significant difference for HairF (6-year-old children [9]). Regarding birth mode, studies show that neonates born vaginally exhibit higher HairF and HairE than those born via cesarean section (C-section) up to 10 days postnatally [30] and that birth complications are associated with lower HairE and a higher HairF to HairE ratio [31]. To our knowledge, the effects of these variables beyond the first year of life have not been investigated. The same applies to the APGAR score, an established neonatology tool assessing term newborns' postnatal adaptation at one, five, and 10 min based on five parameters (i.e., appearance, pulse, grimace, activity, respiration, [32]).

Finally, previous research considered potential effects of exposure to the COVID-19 pandemic on stress-related biomarkers in hair, showing for instance a positive association between HairF and the presence of pandemic-related stressors, such as parental job loss and social distancing, in older children aged 5**–**14 years [33]. However, studies on children aged 0**–**2 years are lacking.

Despite hair being widely used as a biospecimen for glucocorticoid analysis, research into sex steroids like dehydroepiandrosterone (HairDHEA) and progesterone (HairP) in hair is still emerging [4,34], especially in young children. Initial studies suggest positive associations between HairDHEA and female sex and gestational age in newborns [35]. Research on HairP largely focuses on adult hair samples, for instance reporting higher HairP in summer compared to spring and with increased sunlight exposure [27,36] and lower HairP with longer storage time [29,37].

Taken together, evidence on factors influencing hair glucocorticoids and sex steroid levels in children aged 0**–**2 years remains scarce. Early-life changes in hair characteristics, including natural hair color, may affect steroid concentrations in children under four [20], highlighting the need to examine influences such as age, hair color, and perinatal factors in this age group [18]. As part of a large prospective cohort study including four measurement points from birth to two years of age, the current study had the primary aim to evaluate the effects of *developmental factors* (i.e., sociodemographic and birth-related influences including exploration of the APGAR score), as well as *methodological and contextual factors* (i.e., hair-related, organizational variables and COVID-19 pandemic exposure) on the reliably detected steroid hormones HairF, HairE, HairDHEA, and HairP in young children. A secondary aim was to provide a description of developmental trajectories of the concentrations of these four steroids plus hair testosterone (HairT) and hair corticosterone (HairB) up to two years given rapid physiological developmental changes in this time as well as to investigate cross-sectional associations among the four reliably detected hormones. It was expected that the observed hormonal trajectories would correspond to normative developmental patterns reported in the literature [35,38], with peak levels of HairF, HairE, and HairP expected in samples taken shortly after birth, reflecting partly intrauterine steroid secretion, and HairP exhibiting the highest concentrations of these steroids.

## 2 Methods

### 2.1 Study design and participants

The present study is part of the prospective multi-method cohort study “Dresden Study on Parenting, Work, and Mental Health” (DREAM) which currently comprises seven assessment points spanning from pregnancy (T1 DREAM) to childhood (T7 DREAM). It also includes the endocrine sub-study DREAM_HAIR_, which investigates the relationship between long-term steroid hormone and endocannabinoid levels in hair and mental health-related outcomes in mothers, their partners, and their offspring from pregnancy up to 4.5 years after birth. Although endocannabinoids (AEA, 1AG/2AG, SEA, PEA, OEA) were quantified in the same hair samples, they were not included in the present analyses. General inclusion criteria for participating in DREAM included: current pregnancy, Dresden residency (Germany), and sufficient German proficiency to complete the questionnaires. For DREAM_HAIR_, exclusion criteria for children included serious diseases (e.g., craniotomy, cerebellar hemorrhage, tachycardia). For details on the DREAM and DREAM_HAIR_ study, see Ref. [39].

**Fig. 1:**
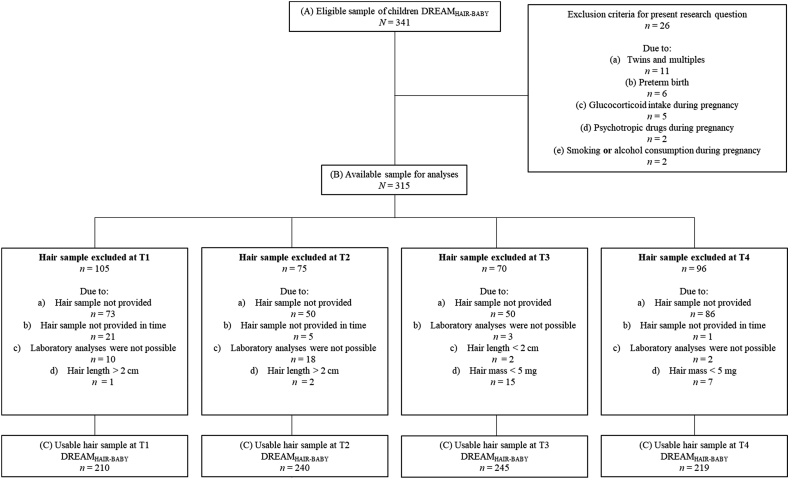
Flowchart of retention rate and exclusion criteria resulting in the final hair steroid sample. *Note.* T1 DREAM_HAIR-BABY_ = within the first 3 weeks after birth. T2 DREAM & DREAM_HAIR-BABY_ = 8 weeks after birth. T3 DREAM_HAIR-BABY_ = 14 months after birth. T4 DREAM_HAIR-BABY_ = two years after birth. The high number of missing hair samples at T1 (*n* = 73) is due to the absence of scalp hair in newborns. Data until 10th of March 2023 (version 10 of the quality-assured data files, prospective data collection was complete for T1**–**T4 and ongoing for T5**–**T7).

For this investigation, questionnaire-derived data, hair samples and hair-related questionnaires were obtained at T1 (DREAM: during pregnancy; DREAM_HAIR-BABY_: within three weeks after birth), T2 (eight weeks after birth), T3 (14 months after birth), and T4 (two years after birth). Exclusion criteria for this study included being a twin or multiple, preterm birth (<37th week of pregnancy), maternal glucocorticoid or psychotropic drug intake, smoking, or alcohol consumption during pregnancy. Hair samples were excluded if the child had taken glucocorticoids within three months prior to assessment, if samples were not provided within a defined time frame, if hair length or mass was unsuitable (hair length: >2 cm (T1, T2), <2 cm (T3, T4); hair mass <5 mg (T3, T4), or laboratory analyses were not feasible (see Fig. 1 for final sample sizes). The exclusion criterion of hair samples <2 cm was not applied at T1 and T2, to be able to capture intrauterine and early postpartum steroid secretion in a sufficiently large sample. However, hair samples >2 cm at T1 were excluded because longer segments would reflect earlier intrauterine periods beyond the 3rd trimester and, given substantial hormonal changes during pregnancy, could reduce comparability across participants. At T2, samples >2 cm were also excluded to avoid overlapping time windows between T1 and T2 assessments as well as longer segments also reflecting in part intrauterine levels rather than purely postpartum levels as intended. Sample sizes were as follows: *n* = 210 (T1), *n* = 240 (T2), *n* = 245 (T3), and *n* = 219 (T4).

The Ethics Committee of TUD Dresden University of Technology granted ethical approval for the study (No: EK 278062015) and written informed consent was obtained from all participants for their and their children's participation in the DREAM and DREAM_HAIR_ studies, separately, in accordance with the Declaration of Helsinki.

### 2.2 Measurements

***Sociodemographic measures*** were derived from the basic DREAM study. Parents reported their children's sex at T2 DREAM and weight at each subsequent measurement point (T2–T4 DREAM). The weight reported at T2 DREAM corresponds to the birth weight. Regarding maternal sociodemographic characteristics, information on age, marital status, parity, and academic degree were assessed at T1 DREAM.

***Birth-related measures*** were assessed as part of DREAM, based on maternity records from the “Mutterpass”. Parents provided information about their children regarding gestational week, birth mode (unassisted vaginal birth, instrumental vaginal birth, planned C-section, or unplanned C-section), and the APGAR score (recorded 5 min after birth; T2 DREAM).

***Hair-related measures*** were obtained through an in-house hair protocol [40] administered to parents, which assessed hair-related characteristics (e.g., hair washing frequency, natural hair color, sunlight exposure per week) and health-related factors (e.g., glucocorticoid intake three months prior to the measurement) of their children (T1**–**T4 DREAM_HAIR-BABY_). Parents provided free-text descriptions of natural hair color, which study staff subsequently dichotomized into light (blond, dark blond, red blond) and dark (red, light brown, brown, dark brown, black), yielding data of the participating children at each assessment point (T1–T4 DREAM_HAIR-BABY_). Season at time of hair sampling (hereafter referred to as ‘season’) was determined based on sample collection date. Four categories were defined according to meteorological seasons: winter (December-February), spring (March-May), summer (June-August), and fall (September-November).

***Organizational measures*** concerning storage time was derived from DREAM_HAIR_ and determined by calculating the weeks between date of hair sample collection and date of laboratory analysis (T1**–**T4 DREAM_HAIR-BABY_).

***COVID-19 pandemic***. All participating children were categorized into one of two groups based on their exposure to the COVID-19 pandemic (classified as 'before/after pandemic'), using a dichotomous variable (yes/no; T1**–**T4 DREAM_HAIR-BABY_). For more information, see Appendix A.1.

***Hair steroid analyses***. HairF, HairE, HairDHEA, HairP, HairT, and HairB were quantified in scalp-near 2 cm hair segments collected from the scalp-near posterior vertex position at each assessment point (T1–T4 DREAM_HAIR-BABY_). In most cases, hair samples were collected by parents who received materials for sample collection by mail along with detailed written instructions and a link to a step-by-step instructional video [41], demonstrating the procedure. In a small number of cases, samples were collected by trained study personnel. Given the average hair growth rate of 1 cm per month [42], this segment represents the steroid hormone secretion for the two months prior to hair sampling. Collected hair samples were stored in a dry, dark environment at room temperature wrapped in aluminum foil, and subsequently sent to the biochemical laboratory at TUD Dresden University of Technology (Prof. Clemens Kirschbaum) in five batches. Laboratory analyses were conducted using liquid chromatography-tandem mass spectrometry (LC-MS/MS) according to a validated protocol with high sensitivity, reliability, and specificity [43]. Upon initial analysis of the biomarkers, the high number of non-detectable values for HairT (27.3%–67.8%) and HairB (2.9%–100%) precluded further analysis; therefore, only descriptive results are reported.

### 2.3 Statistical analyses

The research questions and analysis plan of this study were preregistered (https://osf.io/yqgu7), with deviations from the preregistration detailed in Appendix A.2. All analyses were performed using IBM SPSS Statistics 29, while correlations between hair steroids and continuous determinants were calculated using the psych and boot packages in R.4.3.0 and graphs were created using the ggplot2 package in R.4.4.2. Non-detectable values were replaced by the lowest detectable value within the sample divided by two [44]. Hair steroid values were log-transformed to reduce positive skewness. Outliers were replaced by the lowest or highest concentration that could be reliably detected in the sample (i.e., values within mean ± 3 *SD*; [44]). To address potential batch effects—systematic errors that could generate spurious results or obscure the signal of interest—were controlled by regressing batch on the respective hair steroid levels for each time point, with the residuals preserved for subsequent analyses [27,36]. Due to the non-normal distribution of residuals, bootstrapping procedures were employed. Bias-corrected and accelerated (BCa) bootstrap 95% confidence intervals (CIs) were applied to assess statistical significance.

To analyze cross-sectional associations among four hair steroids (i.e., HairF, HairE, HairDHEA, HairP) in children at each time point T1**–**T4, Spearman Rank correlations were calculated. A repeated-measures ANOVA was conducted to assess potential changes in hair steroid concentrations across all time points T1**–**T4. To address the primary research questions, the following analyses were conducted with hair steroids (i.e., HairF, HairE, HairDHEA, HairP) at each time point (T1**–**T4) as the outcome variable: First, for all continuous determinants, correlations were computed respectively between each independent variable (i.e., age, weight, hair washing frequency, sunlight exposure, storage time, gestational week, APGAR score) and each hair steroid. Second, for dichotomous and categorial determinants (i.e., sex, natural hair color, season, birth mode, COVID-19 pandemic exposure), a *t*-test or a one-way Welch Analysis of Variance (ANOVA) was conducted to investigate their association with each hair steroid. Finally, determinants significant in the previous analyses were included as predictors to estimate their impact on each hair steroid (outcome) for the corresponding time point in a multiple regression analysis. To this end, categorical determinants with more than two categories were dummy-coded with a reference category (i.e., season: winter; birth mode; unassisted vaginal birth). Due to multiple testing, statistical correction was applied to control for inflated type 1 error. For each predictor representing one hypothesis (12 predictors), we applied the Šidák correction for *m* = 16 comparisons (four steroids × four time points). Using the formula 1 − (1 − α)^1/m^, the adjusted significance threshold was set at *p* = .003, with values below this threshold considered statistically significant. While multiple testing correction may reduce power to detect true effects [45,46], results with *p* < .05 were retained for predictors to be included in regression analyses in line with the exploratory nature of this study focusing on the narrow developmental timeframe of the first two years of life.

## 3 Results

### 3.1 Descriptive statistics

**Fig. 2:**
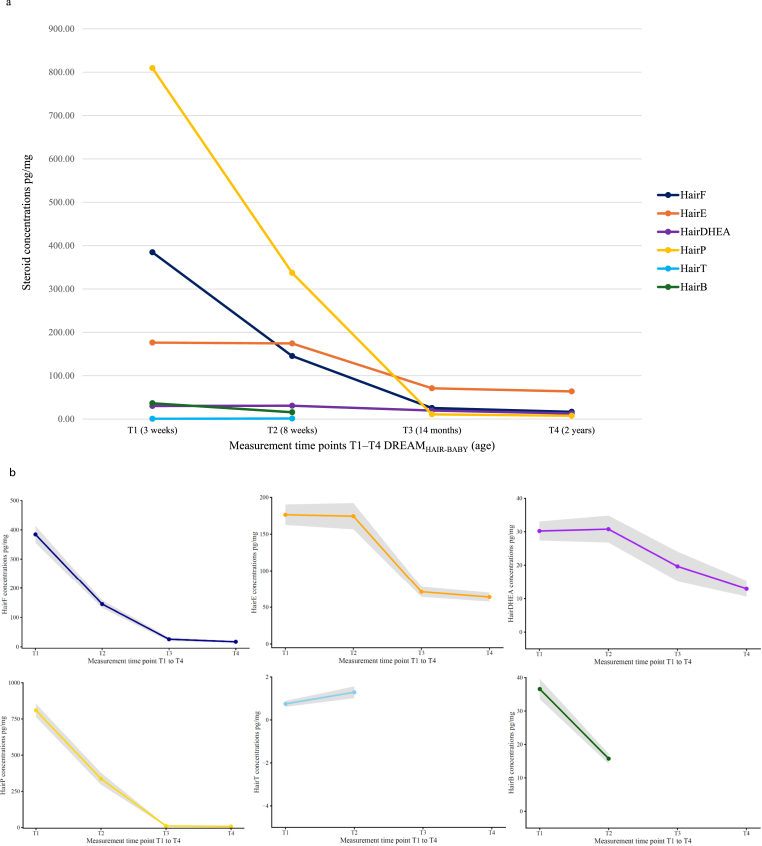
Trajectories of hair steroid concentrations during the first two years of life: a) without and b) with confidence bands. HairF = hair cortisol; HairE = hair cortisone; HairDHEA = hair dehydroepiandrosterone; HairP = hair progesterone; HairT = hair testosterone; HairB = hair corticosterone. Note: HairT and HairB levels at T3 and T4 were not included, due to the high proportion of non-detectable values (>60 %).

Table 1 presents the characteristics of the participating children and their mothers, including selected hair and birth-related parameters. All children in this study were born at term (*M =* 40.41 ± 1.06 weeks), with 80.3% being born by unassisted vaginal birth. Postnatal adaptation was predominantly favorable, as indicated by a mean APGAR score of 9.51 ± 1.01. Table 2 presents descriptive statistics of all hair steroids (HairF, HairE, HairDHEA, HairP, HairT, HairB) across the two-year postnatal period (T1**–**T4 DREAM_HAIR-BABY_). Considering absolute mean levels relative to other steroids, HairP, HairF, and HairE exhibited the highest concentrations at T1. At T2, HairP showed the highest levels, while at T3 and T4, HairE exhibited the highest concentrations. As shown in Fig. 2a, HairF, HairE, HairDHEA and HairP showed a decline over the four measurement points. These differences in steroid concentrations over time were statistically confirmed by repeated-measures ANOVA (see Appendix B). For HairDHEA, HairP, HairT, and HairB, the proportion of non-detectable values increased significantly over time, with the highest proportions observed for HairB at T4 (100%), HairT at T3 (67.76%), HairDHEA at T4 (20.09%), and HairP at T4 (11.42%). For further details, see Table 2.

**Table 1:** Sample characteristics of children (*N* = 315) and mothers (*N* = 309)^a^.

Sample characteristics	*n* (%) or *Mean* ± *SD* (*Range*)
***Children***	
**Sociodemographic variables**	
Sex^c^	
Female (*n*, %)	132 (57.1)
Male (*n*, %)	99 (42.9)
Age	
T1 (in days; *M*, *SD*, *Range*)^f^	9.61 ± 6.16 (0–21)
T2 (in weeks; *M*, *SD*, *Range*)^g^	8.40 ± 1.24 (7–14)
T3 (in months; *M*, *SD*, *Range*)^h^	13.89 ± .62 (12–16)
T4 (in months; *M*, *SD*, *Range*)^i^	23.88 ± .59 (23–26)
Weight	
T2 (g; M, SD, Range)^c^^,^^k^	3409.63 ± 456.29 (2390–4890)
T3 (kg; *M*, *SD, Range*)^d^	9.35 ± 1.10 (6.01–12.44)
T4 (kg; *M*, *SD*, *Range*)^e^	12.15 ± 1.35 (8.5–16)
	
**Hair-related variables**	
Hair washing frequency per week	
T1 (*M*, *SD*, *Range*)^f^	.21 ± .48 (0–3)
T2 (*M*, *SD*, *Range*)^g^	1.45 ± 1.03 (0–7)
T3 (*M*, *SD*, *Range*)^h^	1.80 ± 1.2 (0–7)
T4 (*M*, *SD*, *Range*)^i^	1.66 ± 1.08 (0–7)
Natural hair color T1^f^	
Light (*n*, %)	62 (32.8)
Dark (*n*, %)	127 (67.2)
Natural hair color T2 g	
Light (*n*, %)	94 (43.9)
Dark (*n*, %)	120 (56.1)
Natural hair color T3^h^	
Light (*n*, %)	180 (80.4)
Dark (*n*, %)	44 (19.6)
Natural hair color T4^i^	
Light (*n*, %)	167 (80.7)
Dark (*n*, %)	40 (19.3)
Sunlight exposure in minutes/week	
T1 (*M*, *SD*, *Range*)^f^	20.09 ± 55.41 (0–630)
T2 (*M*, *SD*, *Range*)^g^	161.23 ± 239.68 (0–1800)
T3 (*M*, *SD*, *Range*)^h^	262.59 ± 350.16 (19–2220)
T4 (*M*, *SD*, *Range*)^i^	317.02 ± 396.5 (27–2520)
Season at time of hair sampling T1^f^	
Winter, Dec-Feb (*n*, %)	44 (21.0)
Spring, Mar-May (*n*, %)	61 (29.0)
Summer, Jun-Aug (*n*, %)	56 (26.7)
Fall, Sep-Nov (*n*, %)	49 (23.3)
Season at time of hair sampling T2^g^	
Winter, Dec-Feb (*n*, %)	53 (22.1)
Spring, Mar-May (*n*, %)	70 (29.2)
Summer, Jun-Aug (*n*, %)	68 (28.3)
Fall, Sep-Nov (*n*, %)	49 (20.4)
Season at time of hair sampling T3^h^	
Winter, Dec-Feb (*n*, %)	51 (20.8)
Spring, Mar-May (*n*, %)	63 (25.7)
Summer, Jun-Aug (*n*, %)	80 (32.7)
Fall, Sep-Nov (*n*, %)	51 (20.8)
Season at time of hair sampling T4^i^	
Winter, Dec-Feb (*n*, %)	47 (21.5)
Spring, Mar-May (*n*, %)	58 (26.5)
Summer, Jun-Aug (*n*, %)	57 (26.0)
Fall, Sep-Nov (*n*, %)	57 (26.0)

**Organizational variable**	
Storage time in weeks	
T1 (*M*, *SD*, *Range*)^f^	47.07 ± 15.67 (16–115)
T2 (*M*, *SD*, *Range*)^g^	39.97 ± 14.25 (9–70)
T3 (*M*, *SD*, *Range*)^h^	35.72 ± 13.60 (7–64)
T4 (*M*, *SD*, *Range*)^i^	35.00 ± 14.96 (6–63)

**Birth-related variables**	
Gestational age (in weeks; *M*, *SD*, *Range*)^c^	40.41 ± 1.06 (38–42)
Birth mode^c^	
Unassisted vaginal birth (*n*, %)	184 (80.3)
Instrumental vaginal birth (*n*, %)	15 (6.6)
Planned cesarean section (*n*, %)	15 (6.6)
Unplanned cesarean section (*n*, %)	15 (6.6)
APGAR score, 2nd value taken 5 min after birth (*M*, *SD*, *Range*)^c^	9.51 ± 1.01 (0–10)

**COVID-19 pandemic exposure**	
COVID-19 pandemic exposure T1^f^	
Before/after pandemic (*n*, %)	159 (75.7)
COVID-19 pandemic exposure T2^g^	
Before/after pandemic (*n*, %)	171 (71.3)
COVID-19 pandemic exposure T3^h^	
Before/after pandemic (*n*, %)	72 (29.4)
COVID-19 pandemic exposure T4^i^	
Before/after pandemic (*n*, %)	5 (2.3)
	
***Mothers* Sociodemographic variables**^j^	
Age (in years; *M*, *SD*, *Range*)^b^	30.24 ± 3.93 (18–42)
In a romantic relationship (*n*, %)^b^	305 (99.9)
Primiparous (*n*, %)^b^	254 (82.2)
Academic degree (*n*, %)^b^	197 (64.0)

Note.

a Total *n* varies slightly due to missing values.

b T1 DREAM (*n* = 309; *M* = 26.75 pregnancy week, *SD* = 5.45, *Range* = 11–40).

c T2 DREAM (*n* = 261; *M* = 8.35 weeks after birth date, *SD* = 1.26, *Range* = 6–14).

d T3 DREAM (*n* = 238; *M* = 13.74 months after birth date, *SD* = .58, *Range* = 13-16).

e T4 DREAM (*n* = 208; *M* = 23.87 months after birth date, *SD* = .47, *Range* = 23-25).

f T1 DREAM_HAIR-BABY_ (*n* = 210; *M* = 10.22 days after birth, *SD* = 4.11, *Range* = 0–21).

g T2 DREAM_HAIR-BABY_ (*n* = 240; *M* = 8.40 weeks after birth, *SD* = 1.24, *Range* = 7–14).

h T3 DREAM_HAIR-BABY_ (*n* = 245; *M* = 13.89 months after birth, *SD* = .62, *Range* = 12–16).

i T4 DREAM_HAIR-BABY_ (*n* = 219; *M* = 23.88 months after birth, *SD* = .59, *Range* = 23–26).

j All mothers with a child participating at any time point at DREAM _HAIR-BABY_ were included in sociodemographic analysis.

k Based on the birth weight recorded in the official maternity records and provided by maternal self-report.

**Table 2:** Descriptive statistics of hair steroid concentrations.

Hair steroid concentrations (pg/mg)	*Mean* ± *SD* (*Range*)	Non-Detectables *n* (%)
**Hair cortisol**		
HairF (T1; *M*, *SD*, *Range*)^a^	384.64 ± 212.61 (3.26–1042.88)	0
HairF (T2; *M*, *SD*, *Range*)^b^	145.35 ± 105.43 (23.99–618.63)	0
HairF (T3; *M*, *SD*, *Range*)^c^	25.53 ± 49.47 (.79–340.58)	0
HairF (T4; *M*, *SD*, *Range*)^d^	16.82 ± 16.86 (.44–120.61)	0
**Hair cortisone**		
HairE (T1; *M*, *SD*, *Range*)^a^	176.41 ± 102.60 (30.58–574.11)	0
HairE (T2; *M*, *SD*, *Range*)^b^	174.45 ± 140.41 (20.82–1146.27)	0
HairE (T3; *M*, *SD*, *Range*)^c^	71.01 ± 56.10 (4.19–338.46)	0
HairE (T4; *M*, *SD*, *Range*)^d^	63.84 ± 45.93 (1.24–179.04)	0
**Hair dehydroepiandrosterone**		
HairDHEA (T1; *M*, *SD*, *Range*)^a^	30.24 ± 20.92 (6.99–168.87)	2 (.96)
HairDHEA (T2; *M*, *SD*, *Range*)^b^	30.80 ± 31.89 (1.67–208.48)	4 (1.67)
HairDHEA (T3; *M*, *SD*, *Range*)^c^	19.65 ± 35.17 (.24–436.30)	33 (13.47)
HairDHEA (T4; *M*, *SD*, *Range*)^d^	12.99 ± 17.67 (.67–151.36)	44 (20.09)
**Hair progesterone**		
HairP (T1; *M*, *SD*, *Range*)^a^	809.37 ± 335.86 (.35–1763.52)	0
HairP (T2; *M*, *SD*, *Range*)^b^	337.23 ± 322.65 (3.29–1645.69)	0
HairP (T3; *M*, *SD*, *Range*)^c^	10.98 ± 33.58 (.01–291.96)	13 (5.31)
HairP (T4; *M*, *SD*, *Range*)^d^	7.62 ± 21.47 (.04–146.05)	25 (11.42)
**Hair testosterone**^e^		
HairT (T1; *M*, *SD*, *Range*)^a^	.75 ± 1.0 (.04–5.98)	57 (27.27)
HairT (T2; *M*, *SD*, *Range*)^b^	1.29 ± 1.58 (.09–8.78)	104 (43.51)
HairT (T3; *M*, *SD*, *Range*)^c^	.84 ± 1.43 (.03–7.59)	166 (67.76)
HairT (T4; *M*, *SD*, *Range*)^d^	.77 ± 1.07 (.03–4.85)	134 (61.19)
**Hair corticosterone**^e^		
HairB (T1; *M*, *SD*, *Range*)^a^	36.61 ± 22.31 (3.86–99.84)	6 (2.87)
HairB (T2; *M*, *SD*, *Range*)^b^	15.77 ± 12.07 (2.41–62.25)	50 (20.92)
HairB (T3; *M*, *SD*, *Range*)^c^	Not detectable	243 (99.59)
HairB (T4; *M*, *SD*, *Range*)^d^	Not detectable	219 (100)

Note. HairF = hair cortisol; HairE = hair cortisone; HairDHEA = hair dehydroepiandrosterone; HairP = hair progesterone; HairT = hair testosterone; HairB = hair corticosterone.

a T1 DREAM_HAIR-BABY_ (*n* = 210; *M* = 10.22 days after birth, *SD* = 4.11, *Range* = 0–21).

b T2 DREAM_HAIR-BABY_ (*n* = 240; *M* = 8.40 weeks after birth, *SD* = 1.24, *Range* = 7–14).

c T3 DREAM_HAIR-BABY_ (*n* = 245; *M* = 13.89 months after birth, *SD* = .62, *Range* = 12–16).

d T4 DREAM_HAIR-BABY_ (*n* = 219; *M* = 23.88 months after birth, *SD* = .59, *Range* = 23–26).

e Due to the large number of non-detectable values, these data were not included in the main analyses.

### *3*.2 Cross-sectional associations between HairF, HairE, HairDHEA, and HairP (T1–T4 DREAM_HAIR-BABY_)

HairF and HairE were moderately associated at T1 (*r*_*s*_ = .34, *p* < .001), and highly associated at T3 and T4, respectively (*r*_*s*_ = .90, *p* < .001). At T2, no significant relationship was revealed (*r*_s_ = .00, *p* > .05) Moreover, HairF and HairDHEA exhibited weak positive correlations across T1**–**T4 (*r's* ≥ .19; *p's* ≤ .006), as did HairE and HairDHEA at T1, T3, and T4 (*r's* ≥ .15; *p's* ≤ .002). A moderate (T2), but later weak (T4), positive correlation was observed between HairP and HairF (*r's* ≥ .25; *p's* ≤ .001). The correlation between HairP and HairE shifted from moderate negative at T2 (*r's* ≥ −.41; *p's* ≤ .044) to weak positive with an increasing coefficient by the second year of life (*r*_*s*_*'s* ≥ .13; *p's* ≤ .044). In contrast, the positive correlation between HairDHEA and HairP was initially weak (*r's* ≥ .14; *p's* ≤ .027, T2**–**T3) but became moderate over time (*r*_*s*_ = .30, *p* < .001, T4). For further information, refer to Appendix C.

### 3.3 Cross-sectional associations between hair steroids and determinants (T1–T4 DREAM_HAIR-BABY_)

The findings indicated that neither (birth) weight (p's > .189) nor exposure to the COVID-19 pandemic (*p's* > .248) were associated with any hair steroid at any assessed time point. Besides a significant correlation between the APGAR score and HairE at T1 (*r*_*s*_ = −.21, *p* = .038), no significant associations were observed between the APGAR score and the remaining hair steroids at any given time point (*p's* > .294). Significant results for the remaining determinants will be presented for each steroid. A detailed presentation of the results can be found in Appendix D–I.

#### 3.3.1 Associations with HairF

***Developmental factors: Sociodemographic determinants.*** HairF showed a negative association with children's age at T1 (*r*_*s*_ = −.24, *p* = .012) and a nearly significant association at T2 (*r*_*s*_ = −.18, *p* = .050), with the strength of the association decreasing over time. ***Birth-related determinants.*** Gestational week was positively associated with HairF at both T1 (*r*_*s*_ = .27, *p* = .008) and T2 (*r*_*s*_ = .21, *p* = .023). Finally, there were significant differences regarding birth mode at T1 (*F*(3, 22.34) = 7.87, *p* < .001) and T2 (*F*(3,224) = 5.42, *p* = .001). Post-hoc analyses revealed that at T1, HairF was higher when the child was born by unassisted (*M*_*Diff*_ = 157.83, *p* = .002) or instrumental vaginal birth (*M*_*Diff*_ *= *321.58, *p* = .020) compared to planned C-section. At T2, instrumental vaginal birth was associated with higher HairF compared to unassisted vaginal birth (*M*_*Diff*_ = .20, *p* = .027) or planned C-section (*M*_*Diff*_ = .37, *p* < .001).

***Methodological and contextual factors: Hair-related determinants.*** Season was significantly associated with HairF at T3 and T4. At T3, higher HairF was observed in winter (*M*_*Diff*_ = .27, *p* = .014), summer (*M*_*Diff*_ = .27, *p* = .005), and fall (*M*_*Diff*_ = .24, *p* = .036) compared to spring, while at T4, higher HairF was observed in summer (*M*_*Diff*_ = .30, *p* = .003) and fall (*M*_*Diff*_ = .36, *p* < .001) compared to spring. ***Organizational determinant.*** Storage time was negatively associated with HairF at T4 (*r*_*s*_ = −.26, *p* = .006), with no significant effect at the other measurement points T1**–**T3 (*p's* > .80).

#### 3.3.2 Associations with HairE

***Developmental factors: Birth-related determinants.*** In exploratory correlation analyses, a higher APGAR score was associated with lower HairE at T1 (*r*_*s*_ = −.21, *p* = .038).

***Methodological and contextual factors: Hair-related determinants.*** HairE was negatively associated with hair washing frequency at T2 (*r*_*s*_ = −.24, *p* = .010). Additionally, season was associated with HairE at T3 and T4. At T3, post-hoc analyses revealed higher HairE in winter (*M*_*Diff*_ = .20, *p* = .021), summer (*M*_*Diff*_ = .23, *p* = .001), and fall (*M*_*Diff*_ = .21, *p* = .012) compared to spring. At T4, higher HairE was observed in fall compared to winter (*M*_*Diff*_ = 26.72, *p* = .015) as well as in summer (*M*_*Diff*_ = 28.35, *p* < .001) and fall (*M*_*Diff*_ = 34.14, *p* < .001) compared to spring. ***Organizational determinant.*** Storage time was negatively associated with HairE only at T4 (*r*_*s*_ = −.30, *p* = .001), with no significant effect at the other measurement points T1–T3 (*p's* > .122).

#### 3.3.3 Associations with HairDHEA

***Developmental factors: Sociodemographic determinants.*** HairDHEA was lower in female children compared to males at T4 (*t* = −2.64, *p* = .009).

***Methodological and contextual determinants: Hair-related determinants.*** Sunlight exposure was positively associated with HairDHEA at T3 (*r*_*s*_ = .19, *p* = .034) and T4 (*r*_*s*_ = .20, *p* = .038) and season was associated with HairDHEA at T3 (*F*(3,241) = 9.72, *p* < .001) and T4 (*F*(3,215) = 8.99, *p* < .001). Specifically, at T3, HairDHEA was higher in summer compared to winter (*M*_*Diff*_ = .30, *p* = .049) and higher in both summer (*M*_*Diff*_ = .53, *p* < .001) and fall (*M*_*Diff*_ = .49, *p* < .001) compared to spring. At T4, HairDHEA was elevated in summer (compared to winter: *M*_*Diff*_ = .40, *p* < .001; and spring: *M*_*Diff*_ = .30, *p* = .008) and fall (compared to winter: *M*_*Diff*_ = .40, *p* < .001; and spring: *M*_*Diff*_ = .30, *p* = .010). ***Organizational determinant.*** Storage time was negatively associated with HairDHEA at T4 (*r*_*s*_ = −.26, *p* = .006), with no significant effect at the other measurement points T1–T3 (*p's* > .059).

#### 3.3.4 Associations with HairP

***Developmental factors: Sociodemographic determinants.*** Female children exhibited higher HairP than male children at T1 (*t* = 2.12, *p* = .035) and T2 (*t* = 2.72, *p* = .007).

***Methodological and contextual factors: Hair-related determinants.*** Natural hair color demonstrated significant associations with HairP across T1–T3 (*p's* ≤ .025). At T1 and T2, HairP was higher in children with dark hair compared to those with light hair (T1: *t* = −3.09, *p* = .002; T2: *t* = −2.68, *p* = .008). In contrast, at T3, HairP was higher in children with light hair compared to those with dark hair (*t* = 2.26, *p* = .025). Season was associated with HairP at T1 (*F*(3,205) = 3.22, *p* = .024) and T2 (*F*(3, 122.30) = 3.34, *p* = .022). Post-hoc analyses revealed that at both time points HairP was higher in spring compared to summer (T1: *M*_*Diff*_ = 174.56, *p* = .025; T2: *M*_*Diff*_ = .26, *p* = .050).

#### 3.3.5 Results from regression analysis for hair steroids

Each hair steroid (HairF, HairE, HairDHEA, HairP) was evaluated as a dependent variable for the corresponding time point (T1–T4) in a regression analysis. The determinants identified as significant in the aforementioned analyses were incorporated as predictors. This allowed for the estimation of the determinants' impact on the outcome, with results as follows (see Table 3):

**Table 3:** Multiple regression analyses predicting HairF, HairE, HairDHEA, and HairP.

Variable		T1^g^			T2^h^			T3^i^			T4^j^		
		*β*	*[95% BCa CI]*	*p*^a^	*β*	*[95% BCa CI]*	*p*^a^	*β*	*[95% BCa CI]*	*p*^a^	*β*	*[95% BCa CI]*	*p*^a^
**HairF**													

Age		−.11	[-9.55, .08]	.133	−.13	[-.06, .003]	.067						
Season^b^													
	Spring										−.14	[-.43, .09]	.245
	Summer										.10	[-.11, .33]	.283
	Fall										.14	[-.04, .37]	.115
Storage time											−.05	[-.01, .01]	.658
Gestational week		**.24**	**[19.09, 77.83]**	**.003**^j^	.11	[-.003, .05]	.084						
Birth mode^c^													
	Instrumental vaginal birth	.13	[-41.40, 289.55]	.165	**.17**	**[.05, .31]**	**.009**^j^						
	Planned C- section	**−.13**	**[-169.57,** **−56.64]**	**<.001**	**−.13**	**[-.25, -.03]**	**.016**^j^						
	Unplanned C-section	−.09	[-195.27, 76.96]	.257	−.001	[-.17, .16]	.994						

**HairE**													

Season^b^													
	Spring										−.03	[-18.06, 12.68]	.698
	Summer										**.19**	**[1.19, 36.18]**	**.022**^j^
	Fall										.18	[-2.66, 38.88]	.091
Storage time											−.16	[-1.02, .05]	.068

**HairDHEA**													

Sex^d^											**.15**	**[.02, .3]**	**.023**^j^
Sunlight exposure								.001	[.00, .00]	.989	.12	[.00, .00]	.102
Season^b^													
	Spring							−.14	[-.43, .01]	.064	.13	[-.02, .33]	.088
	Summer							**.21**	**[.08, .52]**	**.012**^j^	**.26**	**[.12, .50]**	**.002**
	Fall							**.16**	**[-.004, .52]**	**.04**^f^	**.21**	**[.05, .46]**	**.012**^j^
Storage time											**−.16**	**[-.01, .00]**	**.038**^j^

**HairP**													

Sex^d^		−.10	[-154.59, 29.59]	.194	−.13	[-.32, −.01]	.060						
Natural hair color^e^		**.20**	**[26.34, 236.50]**	**.012**^j^	**.18**	**[.06, .37]**	**.008**^j^						

Season^b^													
	Spring	.01	[-129.76, 146.54]	.956	.03	[-.16, .25]	.728						
	Summer	−.11	[-187.12, 34.06]	.234	−.12	[-.41, .08]	.173						
	Fall	−.01	[-122.13, 117.84]	.916	−.12	[-.42, .06]	.140						

Note. T1 DREAM_HAIR-BABY_ (*M* = 10.22 days after birth, *SD* = 4.11, *Range* = 0–21). T2 DREAM_HAIR-BABY_ (*M* = 8.40 weeks after birth, *SD* = 1.24, *Range* = 7–14). T3 DREAM_HAIR-BABY_ (*M* = 13.89 months after birth, *SD* = .62, *Range* = 12–16). T4 DREAM_HAIR-BABY_ (*M* = 23.88 months after birth, *SD* = .59, *Range* = 23–26). *β* = Standardised beta coefficient. *95% BCa CI* = 95% bias-corrected and accelerated bootstrap confidence interval (2000 iterations). HairF = hair cortisol; HairE = hair cortisone; HairDHEA = hair dehydroepiandrosterone; HairP = hair progesterone. Significant associations (*p <* .05, two-tailed) presented in bold.

^f^*R*_*adj*_^*2*^*:* atT1 = .125; atT2 = .081; atT4 = .062.

^g^*R*_*adj*_^*2*^: atT4 = .105.

^h^*R*_*adj*_^*2*^: atT3 = .089; at T4 = .134.

^i^*R*_*adj*_^*2*^: at T1 = .047; at T2 = .057.

a Bootstrapped *p* values are reported.

b Reference category is Winter.

c Reference category is Unassisted Vaginal Birth.

d Reference category is Female Sex.

e Reference category is Light Hair Color (blond, dark blond, red-blond).

f Did not survive Šidák correction.

g *n* = 209.

h *n = 239*.

i *n = 245*.

j *n* = 219.

For *HairF*, the following determinants remained significant: Gestational week at T1 (*β* = .24, *p* = .003) and birth mode at T1 (planned C-section: *β* = −.13, *p* < .001) and T2 (instrumental vaginal birth:

*β* = .17, *p* = .009; planned C-section: *β* = −.13, *p* = .016). Planned C-section presented lower HairF compared to unassisted or instrumental vaginal birth at T1 and T2, while instrumental vaginal birth was associated with higher HairF compared to unassisted vaginal birth or planned C-section at T2.

Concerning *HairE*, the season remained significant at T4 with higher concentrations in summer compared to winter (*β* = .19, *p* = .022).

Regarding *HairDHEA*, sex, season, and storage time remained significant. HairDHEA was higher in male children compared to females (*β* = .15, *p* = .023) at T4 and in both summer (T3: *β* = .21, *p* = .012; T4: *β* = .26, *p* = .002) and fall (T3: *β* = .16, *p* = .04; T4: *β* = .21, *p* = .012) compared to winter at T3 and T4. Finally, storage time was negatively associated with HairDHEA (*β* = −.16, *p* = .038) at T4.

For *HairP*, the natural hair color remained significant with higher HairP in children with dark hair compared to those with light hair at T1 (*β* = .20, *p* = .012) and T2 (*β* = .18, *p* = .008).

After Šidák correction (adjusted *p*-value <.003), only birth mode at T1 (HairF) and season at T4 (HairDHEA) remained statistically significant.

## 4 Discussion

This study provides new insights into developmental as well as methodological and contextual factors associated with children's hair steroid concentrations by examining a comprehensive panel of hormones in a large sample of children aged 0**–**2 years. Overall, although several determinants showed statistically significant associations, the observed effect sizes were small, indicating that the magnitude of these effects is limited and that the included variables explain only a modest proportion of variance in hair steroid levels. The identified determinants were as follows: Higher **HairF** was related to a younger age (T1), to winter (T3), summer, and fall (T3, T4) compared to spring, a longer gestational duration (T1, T2), shorter storage time (T4), unassisted vaginal or instrumental vaginal birth compared to planned C-section (T1), and instrumental vaginal birth compared to unassisted vaginal or planned C-section (T2). Higher **HairE** was associated with less frequent hair washing (T2), a shorter storage time (T4), lower APGAR score (T1), when assessed in winter (T3), summer (T3, T4), and fall (T3, T4) compared to spring, and fall compared to spring (T4). Higher **HairDHEA** was linked to male sex (T4), longer sunlight exposure, summer compared to winter (T3, T4), fall compared to winter (T4), and summer and fall compared to spring (T3, T4), and shorter storage time (T4). Higher **HairP** was related to female sex (T1, T2), darker (T1, T2) and lighter hair (T3), respectively, and spring compared to summer (T1, T2). It is important to note that, after correction for multiple testing, significant associations were observed only for higher HairF levels following unassisted vaginal or instrumental vaginal birth compared with planned C-section at T1 and for higher HairDHEA levels in summer relative to winter at T4. Still, a detailed discussion of all findings is provided to inform future research endeavors. Overall, the present pattern of results indicates the most pronounced associations with hair steroids in relation to developmental factors, including age-related declines, gestational age and birth mode. In addition, methodological and contextual factors also showed significant but modest associations with hair steroid concentrations. A summary table is provided in Appendix Table J.1.

### 4.1 Descriptives and cross-sectional associations between hair steroids

Regarding absolute concentrations, high levels of HairF, HairE, and HairP in the first three weeks postnatally (T1), with HairP exhibiting the highest postnatal concentrations, align with previous research [30,35,47]. The significant decline in HairF, within the first three months, with values remaining within age-related reference ranges across all four time points align with reference data in children aged 0–2 years published by De Kruijff et al. [22]. This pattern, together with the transition from higher HairF to higher HairE, supports previous findings from the first six postnatal weeks [30,47] and extends them beyond the first half year of life. The current data also document for the first time similar change patterns for HairDHEA, HairP, and HairB. A comparison with reference values from 4 to 11-year-old children (with no available norms for 0–2 years) from the Dresden LABservice [48] reveals notable deviations: HairF, HairE, and HairP exceeded the >95th percentile, supporting the notion of elevated postnatal steroids that decline with increasing age. Female HairDHEA was above this threshold, while male values were below. HairT in 2-year-old children matched those of older children, while no reference values existed for HairB. Taken together, the alignment of the reported trajectories with expected developmental changes provides convergent evidence supporting the use of hair steroid analysis as a promising methodology in developmental research.

These elevated early-life steroid concentrations and their subsequent decline are indicative of perinatal endocrine development. Fetal and maternal glucocorticoid and progesterone production rises substantially during pregnancy, with cortisol supporting organ maturation [e.g., 49]. Maternal cortisol significantly influences fetal levels, although 11β-hydroxysteroid dehydrogenase type 2 (11β-HSD2) limits fetal exposure to ∼20% of maternal cortisol [50]. It is conceivable that high maternal glucocorticoid and progesterone levels toward the end of gestation may be reflected in neonatal hair. This is consistent with previous data showing that neonatal hair cortisol is positively related to maternal cortisol in hair samples collected within 10 days after birth [47]. Animal data further indicate positive associations between maternal and fetal progesterone levels, suggesting that maternal progesterone levels can influence fetal steroid secretion [51]. These findings align with our observations of elevated neonatal hair steroid levels and suggest that the transition from high intrauterine steroid levels to independent postnatal HPA- and HPG-axis regulation may further explain the initially elevated concentrations and their subsequent normalization during early childhood.

For HairT and HairB, many non-detectables were observed, suggesting testosterone is extremely low in early life. HairB decreased up to eight weeks postnatally and was undetectable thereafter, possibly reappearing in adulthood, consistent with previous findings [43]. In humans, corticosterone plays a minor role, primarily serving as an intermediate in aldosterone biosynthesis, but has also been proposed as a potential biomarker of fetal stress. Through conversion by 11β-HSD2 into its inactive form, 11-dehydrocorticosterone, fetal exposure is limited [[52], [53], [54]]. Its presence in neonatal hair warrants further investigation as a potential indicator of early-life glucocorticoid exposure.

Regarding steroid associations, HairF and HairE showed moderate to high positive correlations across three time points (T1, T3, T4), consistent with previous findings [17,26], likely reflecting 11β-HSD2 activation-inactivation [15]. The lack of an association between these steroids at T2 may be related to marked changes in HairF, while HairE remained constant in this time window in our sample. This finding requires replication and clarification in future research. HairDHEA showed weak positive correlations with HairF (T1**–**T4) and HairE (T1, T3, T4). As a stress-responsive hormone, DHEA may counteract cortisol [3] and serve as a steroidogenic precursor [55]. Progesterone, essential for pregnancy [56] and fetal steroid substrates [7], exhibited moderate associations with HairF and HairE from eight weeks postnatally onward. Additionally, its associations with HairDHEA demonstrated a progressive increase in strength over time, indicating temporally sensitive and physiologically meaningful interactions that warrant further investigation.

### 4.2 Associations between determinants and hair steroids

#### 4.2.1 HairF and HairE

##### 4.2.1.1 Developmental factors

***Sociodemographic determinants.*** The lack of sex differences in HairF and HairE during early childhood is in line with previous studies [19,35] and likely reflects the pre-pubertal hormonal environment, where gonadal steroid influences on glucocorticoids remain minimal. However, our data contradict studies including children beyond the first year of life showing higher HairF in boys [9,18]. It is possible that sex differences in hair glucocorticoids become more pronounced beyond age two. Future studies with repeated assessments from birth up to childhood are needed to test this hypothesis.

Consistent with previous studies [30], the negative age-cortisol association during the first eight weeks after birth represents a critical transition period from fetal to independent HPA-axis functioning, with a sharp decrease in HairF over the first eight weeks of life indicating significant cortisol dynamics in the early days of life, as described by De Kruijff et al. [22]. Given that this association is non-significant in older children [9], this pattern suggests that the timing of measurement may be critical, as the strength of the association appears to diminish with age and may re-emerge at later developmental stages [20,22].

Finally, neither birth weight (retrospectively assessed at T2) nor current weight at T3 and T4 were significantly associated with hair glucocorticoids. This finding is partly consistent with a systematic review reporting either no association or higher HairF in obese children [57]. However, this finding contrasts with studies reporting a positive association between birth weight and HairF [30,47].

***Birth-related determinants.*** Gestational age was positively associated with HairF at T1 and T2, consistent with previous studies [30,47]. Also consistent with earlier research, birth mode contributed to HairF, with higher levels at T1 in infants born via unassisted or instrumental vaginal birth compared to planned C-section, possibly due to the physiological (eu)stress of vaginal birth. The persistence of significance after Šidák correction underscores the relevance of the birth mode as a potential determinant. These results suggest that HairF determined in samples within the first three weeks after birth may represent both intrauterine cortisol exposure and short-term birth-related stress [31]. Similar to patterns observed in cord blood cortisol [58], children born via instrumental vaginal birth exhibited higher HairF than those born unassisted at eight weeks. By T2, HairF declined more sharply in the unassisted vaginal birth group, whereas those born via instrumental vaginal birth exhibited prolonged elevations, indicating a sustained stress response possibly linked to birth-related complications or injuries affecting HPA axis regulation.

To our knowledge, this is the first study to investigate the relationship between the APGAR score and hair steroids in children. Previous research has demonstrated higher saliva cortisol concentrations and lower APGAR scores in preterm newborns [59]. Among the examined steroids, only HairE showed a negative association with APGAR scores at T1. Reduced HairE may reflect physiological downregulation of 11β-HSD activity near term, decreasing cortisol-to-cortisone conversion and consequently reduced cortisone incorporation into hair. These findings are preliminary and exploratory. The limited variability and generally high APGAR scores in this largely healthy, full-term cohort may have produced ceiling effects, restricting the interpretability of the observed associations. Additional research with more diverse samples is needed to further shed light on potential relationships with hair steroids.

##### 4.2.1.2 Methodological and contextual determinants

***Hair-related determinants.*** Aligning with previous findings, lower HairE was associated with higher hair washing frequency at T2 (children: [9]; adults: [17]). The selective effect of HairE to hair-washing, contrasting with null-findings with HairF, suggests differential physiochemical properties between both glucocorticoids that affect their retention within the hair matrix. In adults, it may be explained by the wash-out effect [60]. The observed association in minimally-washed infant hair, mostly with mild or no shampoo, points to alternative mechanisms that require further research.

Regarding season, HairF and HairE were higher in summer and fall (and in winter at 14 months of age) compared to spring, which may reflect sensitivity to temperature, fluid intake, humidity, or altered HPA axis activation. However, the exact mechanisms remain under investigation [61]. While similar seasonal patterns have been found in adults [17,24], the delayed emergence in our cohort suggests that seasonal influences on cortisol and cortisone develop progressively after the first year of life.

***Organizational determinant.*** Storage time was found to be negatively associated with HairF and HairE at T4, with a similar non-significant trend at T3 for HairF. This aligns with findings from previous studies in adults reporting also a negative association with HairF [9,25,27,29]. The fact that the effect was absent in the first postnatal time points indicates that early developmental factors initially override methodological influences, with storage effects becoming detectable once glucocorticoid levels stabilize. This developmental variability likely reflects changes in signal-to-noise ratios. Moreover, overall negative associations at earlier time points (T1–T3 DREAM_HAIR-BABY_) for HairF and HairE albeit non-significant, were directionally consistent (ranging between *r* = −.02 and *r* = −.30). Taken together, these findings suggest that storage effects may contribute to the observed patterns, as previously reported for hair-derived analytes [29,62]. However, the underlying mechanisms remain unclear and may involve processes such as analyte degradation or altered extraction efficiency [29]. It is conceivable that age-related differences in hair structure during early development [e.g., transition from vellus to thicker terminal hair [63], may influence susceptibility to such effects, potentially altering how hair samples respond to storage and pre-analytical processing.

***COVID-19 pandemic exposure.*** Contrary to our expectations, we found no association between COVID-19 pandemic exposure and hair glucocorticoids at any analyzed measurement point (a finding replicated for other steroids). These results contrast with previous studies in children [33,64]. While Perry et al. [33] reported higher HairF in the presence of stressors such as family job loss or social isolation, Fung et al. [64] also observed elevated HairF but could not link them to self-reported pandemic-related stressors. Our findings suggest that the COVID-19 pandemic at this early age may not result in alterations in long-term integrated glucocorticoid levels.

#### 4.2.2 HairDHEA

##### 4.2.2.1 Developmental factors

***Sociodemographic determinants.*** Our findings revealed that male children had higher HairDHEA than female children at two years postnatally. This finding contradicts existing literature that reports higher levels in female newborns and 9-year-olds [35,65]. This specific age pattern could reflect transient hormonal fluctuations specific to the developmental stage, however further longitudinal research is needed to confirm the findings.

##### 4.2.2.2 Methodological and contextual factors

***Hair-related determinants.*** Sunlight exposure and season influenced HairDHEA two years postnatally, with higher levels in summer compared to winter (remaining significant after Šidák correction, highlighting its’ relevance as a potential determinant) and both summer and fall compared to spring. These seasonal effects align with adult studies reporting lower levels in winter [36], potentially reflecting a sunlight-dependent cleavage of the sulfate group from DHEA-S, converting it into its active form, DHEA [23].

***Organizational determinant.*** The storage-related degradation pattern resembling that of glucocorticoids suggests shared chemical vulnerabilities between DHEA and other steroids, likely due to enzymatic or oxidative processes over time. However, this finding contrasts with a study of 81 adults reporting storage effects on glucocorticoids, progesterone, and endocannabinoids, but not DHEA [29]. Such inconsistencies highlight the necessity of systematically controlling for storage duration in hair-based steroid analyses of infants and young children up to two years of age.

#### 4.2.3 HairP

##### 4.2.3.1 Developmental factors

***Sociodemographic determinants.*** HairP was higher in female than male children at T1 and T2, suggesting early postnatal emergence of sex-specific steroidogenic patterns. However, our results contrast with absent sex differences reported in newborns [35], requiring further replication.

##### 4.2.3.2 Methodological and contextual factors

***Hair-related determinants.*** Natural hair color was linked to HairP, with higher concentrations observed in dark-haired individuals during the first 14 months postnatally. Given that progesterone can stimulative melanin synthesis [66], these findings might indicate that elevated intrauterine progesterone levels may influence melanin production and be associated with darker hair in newborns. The declining HairP coinciding with lighter hair emergence at T3 suggest parallel normalization of the progesterone and melatonin systems as maternal hormone influence wanes. Finally, season showed significant associations, with higher concentrations in spring than summer, contradicting adult hair studies that reported higher HairP in summer [37].

## 5 Limitations and conclusions

Limitations include the large number of statistical tests conducted. A correction for multiple testing was applied for regression analyses, but not to all tests. Additional limitations comprise the relatively homogeneous sample, the absence of socioeconomic and hair growth measures, and reliance on self-reported data (e.g. weekly sun exposure without accounting for meteorological conditions), potentially limiting generalizability and introducing measurement bias. Nonetheless, our findings suggest that meaningful effects on hair steroid levels may emerge only at higher levels of sun exposure (UV light). Moreover, the coarse pre-/post- and during-pandemic classification may obscure meaningful stress exposure in children up to two years of age and represents another study limitation, highlighting the need for more detailed and systematic investigation of this variable. Future research with larger cohorts will also be essential to replicate these results and deepen our understanding of early-life steroid regulation. While a considerable proportion of children did not provide a hair sample with a minimum hair length of 2 cm at T1 and T2, they were included in the analyses. As a result, variability in children's hair length may have led to differences in the retrospective time window of steroid secretion reflected by the samples. This should be considered a limitation when interpreting the findings.

Together, our findings indicate that perinatal factors are significantly associated with HairF, with gestational age and birth mode consistently contributing to cortisol levels. Children delivered via instrumental vaginal birth exhibited prolonged HairF elevations, suggesting sustained postnatal stress responses that may shape early HPA-axis regulation. Seasonal variations selectively affected HairF, HairE, and HairDHEA, potentially reflecting photoperiodic influences on steroid metabolism. Sex-specific patterns were only found for DHEA (higher levels in males) and HairP (higher levels in females; however, the influence was no longer significant after accounting for further determinants). Notably, no sex differences in HairF were detected during this early developmental window, contrasting with reports in older children. These findings should be interpreted with caution as sex differences were inconsistent across assessment points and showed limited robustness in multivariable models. In this prepubertal age range, sex-specific differences in hair steroid levels appear subtle and developmentally unstable, but may become more pronounced with the onset of puberty [e.g., 67]. In addition, storage time was associated with several hair-based analytes, underscoring the importance of standardized handling and analytical protocols in hair steroid research (see recommendations in [29]).

Despite multiple statistically significant associations, regression models incorporating all predictors yielded small effect sizes, indicating limited external influence on hair steroid concentrations. Importantly, only a few associations remained significant after correction for multiple testing. Overall, findings indicate that hair steroid analysis in children aged 0–2 years shows limited systematic associations with the examined variables, with variance primarily attributable to genuine physiological processes rather than methodological artifacts. While normative physiological transitions cannot be modified, the knowledge of which factors associate with hair steroid levels in this sensitive time is key to adequate statistical adjustment in order to answer pressing research questions on infant stress regulation. Our findings suggest that hair steroid analysis as a measurement approach may be relatively robust against the influencing variables tested in this study, thus highlighting its promising utility as an objective tool for assessing stress-related biomarkers during critical early development.

Taken together, our findings highlight the continuing relevance of Prof. Clemens Kirschbaum's pioneering work in establishing hair steroid assessment. Our results underscore that, in addition to expected age-related changes, birth-related factors and season are associated with hair steroid levels while other methodological and contextual variables exert only a small influence during the first years of life.

## Author note

We have no conflict of interest to disclose.

## Declaration of generative AI and AI-assisted technologies in the manuscript preparation process

During the preparation of this work, the authors used DeepL Write and ChatGPT in order to improve the language and readability of their manuscript. After using these services, the authors reviewed and edited the content as needed and take full responsibility for the content of the published article.

## Funding

This work was supported by the 10.13039/501100001659German Research Foundation (“Deutsche Forschungsgemeinschaft”) under grant numbers GA 2287/4-1, GA 2287/4-2, and GA 2287/4-3.

## CRediT authorship contribution statement

**Esther J. Hutloff:** Conceptualization, Formal analysis, Methodology, Visualization, Writing – original draft. **Isabel Jaramillo:** Conceptualization, Data curation, Investigation, Methodology, Supervision, Writing – review & editing. **Luisa Bergunde:** Conceptualization, Data curation, Investigation, Methodology, Writing – review & editing. **Susan Garthus-Niegel:** Project administration, Supervision, Writing – review & editing. **Susann Steudte-Schmiedgen:** Conceptualization, Project administration, Supervision, Writing – review & editing.

## Declaration of competing interest

The authors declare that they have no known competing financial interests or personal relationships that could have appeared to influence the work reported in this paper.

## Footnotes

Appendix A Supplementary data to this article can be found online at https://doi.org/10.1016/j.cpnec.2026.100354.
