# Supplementary material for: Developmental and environmental determinants of hair steroids in children from birth to two years postnatally: A comprehensive analysis
Source: Compr Psychoneuroendocrinol. 2026 May 27;27:100354. doi: 10.1016/j.cpnec.2026.100354 (PMC13273773; doi:10.1016/j.cpnec.2026.100354)
Supplement: Multimedia component 1 [file mmc1.docx]

**Appendix A. Additional methodological details**

**A.1 Assessment of the determinant ‘COVID-19 pandemic exposure’**

Exposure to the COVID-19 pandemic was categorized into two groups based on the date of hair sample collection at each time point (T1–T4 DREAM_HAIR-BABY_). Participants completing the respective measurement period between March 10, 2020, and January 15, 2023, were assigned to the 'during pandemic' group (=1). All other participants were classified into the 'before/after pandemic' group (=0). This was represented by a dichotomous variable indicating pandemic exposure (yes/no). Both reference dates were based on infection incidence and implemented restrictions in Germany [1,2]. Nationwide non-pharmaceutical interventions began on March 10th, 2020, starting with recommendations to cancel large events, followed by measures such as self-isolation and movement restrictions. As case numbers declined, these measures were gradually relaxed, with January 15th, 2023, selected as the reference date to reflect a substantial reduction in reported infections and restrictive measures.

**A.2 Deviations from Preregistration**

The research questions and analysis plan of this study were preregistered prior to data analysis on the *Open Science Framework*-Platform (OSF) and are available at: <https://osf.io/yqgu7>.

One exploratory research question was excluded after preregistration: “What are the mean concentrations of hair testosterone and corticosterone in children from birth to two years after birth?” Due to a high number of non-detectable values for both hair testosterone and hair corticosterone, further analyses were not feasible. As this exploratory research question was limited to reporting steroid concentrations and lacked sufficient substance to justify a standalone research question, it was subsequently omitted. Steroid concentrations are now presented in the descriptive results section.

As not explicitly specified in the preregistration, standardized regression coefficients (*β*-weights) were reported to quantify the effect sizes of the determinants on hair steroid levels at each measurement time point (T1–T4 DREAM_HAIR-BABY_).

Subsequently, repeated-measures ANOVAs were employed to statistically assess longitudinal changes in steroid levels across the first two years of life.

**Appendix B. Hair steroid concentrations during the first two years of life**

**Table B.1 Results of repeated-measures ANOVA for differences in mean hair steroid concentrations across the first two years of life (T1**–**T4 DREAM_HAIR-BABY_)**

|  | T1^1^ |  | T2^2^ |  | T3^3^ |  | T4^4^ |  |  |  |  |
| --- | --- | --- | --- | --- | --- | --- | --- | --- | --- | --- | --- |
| **Dependent Variable** | ***M*±*SD*** |  | ***M*±*SD*** |  | ***M*±*SD*** |  | ***M*±*SD*** |  | ***F*(*df ^a^*, *df ^b^*)** | ***p*** | ***η²*** |
| HairF | 384.64 ± 212.61 |  | 145.35 ± 105.43 |  | 25.53 ± 49.47 |  | 16.82 ± 16.86 |  | *F*(1.54,217.13) = **322.3** | < .001 | .70 |
| HairE | 176.41 ± 102.60 |  | 174.45 ± 140.41 |  | 71.01 ± 56.10 |  | 63.84 ± 45.93 |  | *F*(2.14,302.34) = **83.16** | < .001 | .37 |
| HairDHEA | 30.24 ± 20.92 |  | 30.80 ± 31.89 |  | 19.65 ± 35.17 |  | 12.99 ± 17.67 |  | *F*(2.59,364.58) = **22.71** | < .001 | .14 |
| HairP | 809.37 ± 335.86 |  | 337.23 ± 322.65 |  | 10.98 ± 33.58 |  | 7.62 ± 21.47 |  | *F*(1.92,270.86) = **460.64** | < .001 | .77 |
| HairT | 0.75 ± 1.0 |  | 1.29 ± 1.58 |  | 0.84 ± 1.43 |  | 0.77 ± 1.07 |  | *F*(1.80,28.81) = 2.98 | .071 | .16 |
| HairB | 36.61 ± 22.31 |  | 15.77 ± 12.07 |  | not detectable |  | not detectable |  | *F*(1,194) = **183.45** | < .001 | .49 |

*Note.* T1 DREAM_HAIR-BABY_ (*M* = 10.22 days after birth, *SD* = 4.11, *Range* = 0–21). T2 DREAM_HAIR-BABY_ (*M* = 8.40 weeks after birth, *SD* = 1.24, *Range* = 7–14).
T3 DREAM_HAIR-BABY_ (*M* = 13.89 months after birth, *SD* = 0.62, *Range* = 12–16). T4 DREAM_HAIR-BABY_ (*M* = 23.88 months after birth, *SD* = 0.59, *Range* = 23–26).
HairF = hair cortisol; HairE = hair cortisone; HairDHEA = hair dehydroepiandrosterone; HairP = hair progesterone; HairT = hair testosterone; HairB = hair corticosterone. Significant associations presented in bold.

^a^ *Df* between the groups.

^b^ *Df* within the groups.

^1^ *n* = 210, *n* varied slightly due to missing data. ^2^ *n* = 240, *n* varied slightly due to missing data. ^3^ *n* = 245. ^4^ *n* = 219.

**Table B.2 Standard errors of hair steroid concentrations across the first two years of life (T1**–**T4 DREAM_HAIR-BABY_)**

| Hair steroid  Time point | HairF | HairE | HairDHEA | HairP | HairT | HairB |
| --- | --- | --- | --- | --- | --- | --- |
| T1 DREAM_HAIR-BABY_^1^ | 14.71 | 7.10 | 1.45 | 23.23 | 0.07 | 1.54 |
| T2 DREAM_HAIR-BABY_^2^ | 6.82 | 9.08 | 2.06 | 20.87 | 0.14 | 0.78 |
| T3 DREAM_HAIR-BABY_^3^ | 3.16 | 3.58 | 2.25 | 2.15 |  |  |
| T4 DREAM_HAIR-BABY_^4^ | 1.14 | 3.10 | 1.19 | 1.45 |  |  |

*Note.* T1 DREAM_HAIR-BABY_ (*M* = 10.22 days after birth, *SD* = 4.11, *Range* = 0–21). T2 DREAM_HAIR-BABY_ (*M* = 8.40 weeks after birth, *SD* = 1.24, *Range* = 7–14).
T3 DREAM_HAIR-BABY_ (*M* = 13.89 months after birth, *SD* = 0.62, *Range* = 12–16). T4 DREAM_HAIR-BABY_ (*M* = 23.88 months after birth, *SD* = 0.59, *Range* = 23–26).
HairF = hair cortisol; HairE = hair cortisone; HairDHEA = hair dehydroepiandrosterone; HairP = hair progesterone; HairT = hair testosterone; HairB = hair corticosterone. Significant associations presented in bold.

^1^ *n* = 210, *n* varied slightly due to missing data. ^2^ *n* = 240, *n* varied slightly due to missing data. ^3^ *n* = 245. ^4^ *n* = 219.

**Appendix C. Cross-sectional correlations between hair steroids at T1–T4** **DREAM_HAIR-BABY_**

**Table C.1 Spearman rank correlations [*95% BCa CI ^a^*] between HairF, HairE, HairDHEA, and HairP (T1–T4 DREAM_HAIR-BABY_)**

|  | Hair steroid | HairF | HairE | | HairDHEA | | HairP | |
| --- | --- | --- | --- | --- | --- | --- | --- | --- |
| T1^1^ | HairF | – | **.34**** | [.20, .47] | **.19**** | [.03, .35] | .03 | [-.12, .18] |
|  | HairE |  | – | | **.25**** | [.11, .38] | -.04 | [-.19, .11] |
|  | HairDHEA |  |  | | – | | .09 | [-.05, .24] |
|  | HairP |  |  | |  | | – | |
| T2^2^ | HairF | – | .00 | [-.12, .13] | **.26**** | [.13, .38] | **.41**** | [.29, .51] |
|  | HairE |  | – | | .01 | [-.12, .14] | **-.41**** | [-.52, -.30] |
|  | HairDHEA |  |  | | – | | **.14*** | [.01, .27] |
|  | HairP |  |  | |  | | – | |
| T3^3^ | HairF | – | **.90**** | [.84, .95] | **.21**** | [.08, .34] | .10 | [-.03, .23] |
|  | HairE |  | – | | **.20**** | [.08, .33] | **.13*** | [-.01, .26] |
|  | HairDHEA |  |  | | – | | **.21**** | [.08, .34] |
|  | HairP |  |  | |  | | – | |
| T4^4^ | HairF | – | **.90**** | [.86, .93] | **.21**** | [.08, .33] | **.25**** | [.12, .38] |
|  | HairE |  | – | | **.15*** | [.03, .28] | **.22**** | [.09, .34] |
|  | HairDHEA |  |  | | – | | **.30**** | [.16, .44] |
|  | HairP |  |  | |  | | – | |

*Note.* T1 DREAM_HAIR-BABY_ (*M* = 10.22 days after birth, *SD* = 4.11, *Range* = 0–21). T2 DREAM_HAIR-BABY_ (*M* = 8.40 weeks after birth, *SD* = 1.24, *Range* = 7–14). T3 DREAM_HAIR-BABY_ (*M* = 13.89 months after birth, *SD* = 0.62, *Range* = 12–16). T4 DREAM_HAIR-BABY_ (*M* = 23.88 months after birth, *SD* = 0.59, *Range* = 23–26). HairF = hair cortisol; HairE = hair cortisone; HairDHEA = hair dehydroepiandrosterone; HairP = hair progesterone. Significant associations (* *p <* .05, two-tailed; ** *p* < .01, two-tailed) presented in bold.

^a^ Values in brackets show the 95% confidence interval (bias-corrected and accelerated bootstrap confidence interval, 2000 iterations) for each correlation.

^1^ *n* = 210, *n* varied slightly due to missing data. ^2^ *n* = 240, *n* varied slightly due to missing data. ^3^ *n* = 245. ^4^ *n* = 219.

**Appendix D. Cross-sectional correlations between potential determinants and hair steroids at T1–T4 DREAM_HAIR-BABY_**

**Table D.1 Spearman rank correlations [*95% BCa CI* ^a^] between determinants and hair steroids in the postnatal period (T1–T4 DREAM_HAIR-BABY_)**

|  | **T1*^1^*** | | | | **T2*^2^*** | | | **T3*^3^*** | | | | **T4*^4^*** | | | |
| --- | --- | --- | --- | --- | --- | --- | --- | --- | --- | --- | --- | --- | --- | --- | --- |
| **Variable** | ***r*** | ***p*** | ***CI*** |  | ***r*** | ***p*** | ***CI*** |  | ***r*** | ***p*** | ***CI*** |  | ***r*** | ***p*** | ***CI*** |
| **HairF** | | | | | | | | | | | | | | | |
| Age | **-.24** | **.012** | [-.36, -.10] |  | -.18 | .050 | [-.30, -.06] |  | -.03 | .448 | [-.16, .11] |  | .10 | .279 | [-.03, .23] |
| Weight | .02 | .762 | [-.12, .16] |  | .05 | .465 | [-.09, .18] |  | -.07 | .337 | [-.21, .05] |  | .00 | .501 | [-.14, .14] |
| Hair washing frequency | -.11 | .231 | [-.22, .02] |  | .02 | .464 | [-.13, .15] |  | -.09 | .265 | [-.22, .03] |  | -.04 | .462 | [-.18, .09] |
| Sunlight exposure | -.12 | .20 | [-.26, .02] |  | -.14 | .117 | [-.28, -.02] |  | -.01 | .499 | [-.15, .11] |  | .00 | .508 | [-.13, .14] |
| Storage time | -.02 | .519 | [-.15, .10] |  | -.02 | .484 | [-.15, .10] |  | -.16 | .080 | [-.28, -.03] |  | **-.26** | **.006** | [-.39, -.13] |
| Gestational week | **.27** | **.008** | [.12, .39] |  | **.21** | **.023** | [.08, .33] |  | .02 | .479 | [-.10, .15] |  | .06 | .398 | [-.07, .18] |
| **HairE** | | | | | | | | | | | | | | | |
| Age | .03 | .468 | [-.12, .17] |  | -.10 | .233 | [-.22, .02] |  | -.05 | .385 | [-.18, .08] |  | .07 | .384 | [-.07, .20] |
| Weight | .07 | .363 | [-.07, .21] |  | .05 | .490 | [-.09, .19] |  | -.06 | .380 | [-.19, .06] |  | -.01 | .505 | [-.15, .13] |
| Hair washing frequency | -.09 | .298 | [-.23, .06] |  | **-.24** | **.010** | [-.36, -.11] |  | -.12 | .163 | [-.25, .00] |  | .00 | .502 | [-.15, .13] |
| Sunlight exposure | .01 | .493 | [-.14, .15] |  | .06 | .360 | [-.07, .19] |  | .03 | .473 | [-.11, .16] |  | .02 | .496 | [-.11, .16] |
| Storage time | -.12 | .181 | [-.25, .00] |  | -.09 | .286 | [-.21, .05] |  | -.13 | .122 | [-.25, -.01] |  | **-.30** | **.001** | [-.42, -.17] |
| Gestational week | .18 | .069 | [.04, .30] |  | .13 | .147 | [.00, .25] |  | .03 | .463 | [-.09, .16] |  | .10 | .238 | [-.03, .24] |
| **HairDHEA** | | | | | | | | | | | | | | | |
| Age | -.07 | .357 | [-.21, .08] |  | -.12 | .196 | [-.25, .01] |  | -.08 | .324 | [-.20, .04] |  | .05 | .419 | [-.08, .18] |
| Weight | .04 | .630 | [-.10, .16] |  | .07 | .288 | [-.07, .20] |  | .02 | .500 | [-.11, .14] |  | -.08 | .334 | [-.21, .07] |
| Hair washing frequency | .08 | .365 | [-.05, .22] |  | -.11 | .229 | [-.23, .02] |  | .02 | .483 | [-.11, .14] |  | -.15 | .104 | [-.29, -.02] |
| Sunlight exposure | -.16 | .121 | [-.30, -.02] |  | -.06 | .405 | [-.17, .07] |  | **.19** | **.034** | [.06, .31] |  | **.20** | **.038** | [.06, .33] |
| Storage time | -.13 | .173 | [-.26, .00] |  | -.04 | .446 | [-.17, .08] |  | -.17 | .059 | [-.29, -.05] |  | **-.26** | **.006** | [-.38, -.12] |
| Gestational week | .02 | .460 | [-.13, .17] |  | .12 | .173 | [.00, .26] |  | .08 | .325 | [-.04, .21] |  | .08 | .333 | [-.06, .22] |
| **HairP** | | | | | | | | | | | | | | | |
| Age | -.03 | .474 | [-.17, .10] |  | -.06 | .383 | [-.19, .08] |  | -.01 | .483 | [-.14, .12] |  | -.05 | .397 | [-.18, .09] |
| Weight | .09 | .189 | [-.06, .24] |  | .07 | .280 | [-.06, .21] |  | -.02 | .484 | [-.16, .10] |  | .07 | .394 | [-.07, .21] |
| Hair washing frequency | .01 | .486 | [-.14, .15] |  | -.09 | .292 | [-.22, .04] |  | -.06 | .387 | [-.19, .06] |  | -.13 | .170 | [-.26, .02] |
| Sunlight exposure | .05 | .420 | [-.11, .19] |  | -.10 | .217 | [-.23, .03] |  | .04 | .458 | [-.08, .16] |  | .05 | .405 | [-.09, .18] |
| Storage time | .05 | .419 | [-.09, .18] |  | .15 | .103 | [.02, .27] |  | -.09 | .289 | [-.22, .05] |  | -.13 | .176 | [-.26, .02] |
| Gestational week | -.08 | .345 | [-.23, .06] |  | .03 | .457 | [-.10, .16] |  | .03 | .486 | [-.09, .14] |  | .09 | .284 | [-.05, .21] |

*Note*. T1 DREAM_HAIR-BABY_ (*M* = 10.22 days after birth, *SD* = 4.11, *Range* = 0–21). T2 DREAM_HAIR-BABY_ (*M* = 8.40 weeks after birth, *SD* = 1.24, *Range* = 7–14). T3 DREAM_HAIR-BABY_ (*M* = 13.89 months after birth, *SD* = 0.62, *Range* = 12–16). T4 DREAM_HAIR-BABY_ (*M* = 23.88 months after birth, *SD* = 0.59, *Range* = 23–26). HairF = hair cortisol;
HairE = hair cortisone; HairDHEA = hair dehydroepiandrosterone; HairP = hair progesterone. Significant associations (*p <* .05, two-tailed) presented in bold.

^a^ Values in brackets show the 95% confidence interval (bias-corrected and accelerated bootstrap confidence interval, 2000 iterations) for each correlation.

^1^ *n* = 174-209. ^2^ *n* = 205-240. ^3^ *n* = 213-245. ^4^ *n* = 178-219. *n* varied due to missing data within each time point.

**Appendix E. Results of *t*-Tests, ANOVAs and post-hoc tests for HairF**

**Table E.1 *t*-Test results for HairF and potential determinants in the postnatal period (T1**–**T4 DREAM_HAIR-BABY_)**

| HairF | | | | | | | | | | | | | | | | |
| --- | --- | --- | --- | --- | --- | --- | --- | --- | --- | --- | --- | --- | --- | --- | --- | --- |
|  |  | T1^1^ | | |  | T2^2^ | | |  | T3^3^ | | |  | T4^4^ | | |
| **Variable** |  | ***M(SD)*** | ***t df)*** | ***p*** |  | ***M(SD)*** | ***t (df)*** | ***p*** |  | ***M(SD)*** | ***t (df)*** | ***p*** |  | ***M(SD)*** | ***t (df)*** | ***p*** |
| Sex |  |  | -.10(195) | .922 |  |  | .32(228) | .751 |  |  | 12(237) | .905 |  |  | .38(211) | .705 |
|  | Female | 22.45 (224.22) |  |  |  | .01 (.27) |  |  |  | .00 (.46) |  |  |  | .02 (.40) |  |  |
|  | Male | 25.36 (183.68) |  |  |  | .00 (.25) |  |  |  | -.01 (.50) |  |  |  | -.01 (.61) |  |  |
| Natural hair color | |  | -1.23(186) | .220 |  |  | -.29(211) | .775 |  |  | .28(222) | .784 |  |  | -1.43(205) | .154 |
|  | Light | -11.97 (178.67) |  |  |  | .00 (.26) |  |  |  | .01 (.47) |  |  |  | -.01 (.45) |  |  |
|  | Dark | 25.91 (207.43) |  |  |  | .01 (.26) |  |  |  | -.01 (.50) |  |  |  | .11 (.67) |  |  |
| COVID-19 pandemic exposure | | | -.19(207) | .849 |  |  | -.02  (237) | .984 |  |  | .45(243) | .656 |  |  | 1.08(217) | .283 |
|  | No | 17.42 (205.77) |  |  |  | .00 (.26) |  |  |  | .02 (.46) |  |  |  | .24 (.13) |  |  |
|  | Yes | 23.77 (201.24) |  |  |  | .00 (.26) |  |  |  | -.01 (.48) |  |  |  | -.01 (.51) |  |  |
| *Note*. T1 DREAM_HAIR-BABY_ (*M* = 10.22 days after birth, *SD* = 4.11, *Range* = 0–21). T2 DREAM_HAIR-BABY_ (*M* = 8.40 weeks after birth, *SD* = 1.24, *Range* = 7–14). T3 DREAM_HAIR-BABY_ (*M* = 13.89 months after birth, *SD* = 0.62, *Range* = 12–16). T4 DREAM_HAIR-BABY_ (*M* = 23.88 months after birth, *SD* = 0.59, *Range* = 23–26). HairF = hair cortisol.  ^1^ *n* = 210, *n* varied slightly due to missing data. ^2^ *n* = 240, *n* varied slightly due to missing data. ^3^ *n* = 245. ^4^ *n* = 219. | | | | | | | | | | | | | | | | |

**Table E.2 ANOVA results for the influence of potential determinants on HairF during the postnatal period (T1**–**T4 DREAM_HAIR-BABY_)**

| HairF | | | | | | | | | | | | | | | | | | | |
| --- | --- | --- | --- | --- | --- | --- | --- | --- | --- | --- | --- | --- | --- | --- | --- | --- | --- | --- | --- |
| **Variable** | T1^1^ | | | |  | T2^2^ | | | |  | T3^3^ | | | |  | T4^4^ | | | |
|  | ***F*** | ***df ^a^*** | ***df ^b^*** | ***Sig*** |  | ***F*** | ***df ^a^*** | ***df ^b^*** | ***Sig*** |  | ***F*** | ***df ^a^*** | ***df ^b^*** | ***Sig*** |  | ***F*** | ***df ^a^*** | ***df ^b^*** | ***Sig*** |
| Season ^c^ | .21 | 3 | 205 | .888 |  | .88 | 3 | 235 | .450 |  | **4.96** | 3 | 241 | .002 |  | **8.19** ^e^ | 3 | 113.72 | <.001 |
| Birth mode ^d^ | **7.87** ^e^ | 3 | 22.34 | <.001 |  | **5.42** | 3 | 224 | 0.001 |  | 1.94 | 3 | 235 | .124 |  | .64 ^e^ | 3 | 26.09 | .597 |

*Note*. T1 DREAM_HAIR-BABY_ (*M* = 10.22 days after birth, *SD* = 4.11, *Range* = 0–21). T2 DREAM_HAIR-BABY_ (*M* = 8.40 weeks after birth, *SD* = 1.24, *Range*
= 7–14). T3 DREAM_HAIR-BABY_ (*M* = 13.89 months after birth, *SD* = 0.62, *Range* = 12–16). T4 DREAM_HAIR-BABY_ (*M* = 23.88 months after birth, *SD* = 0.59, *Range* = 23–26). HairF = hair cortisol. Significant associations presented in bold.

^a^ Df between the groups.

^b^ Df within the groups.

^c^ includes: Winter (reference category), Spring, Summer, Fall.

^d^ includes: Spontaneous vaginal birth (reference category), Instrumental vaginal birth, Planned cesarean section, Unplanned cesarean section.

^e^ WELCH-ANOVA is reported because of inhomogenity of variance.

^1^ *n* = 210, *n* varied slightly due to missing data. ^2^ *n* = 240, *n* varied slightly due to missing data. ^3^ *n* = 245. ^4^ *n* = 219.

**Table E.3 Post-hoc analysis for HairF and potential determinants in the postnatal period (T1**–**T4 DREAM_HAIR-BABY_)**

| HairF | | | | | | | | | | | | | | | | | |
| --- | --- | --- | --- | --- | --- | --- | --- | --- | --- | --- | --- | --- | --- | --- | --- | --- | --- |
|  | |  | T1^1^ | | |  | T2^2^ | | |  | T3^3^ | | |  | T4^4^ | | |
| **Comparison** | |  | ***M_Diff_*** | ***CI*** | ***p*** |  | ***M_Diff_*** | ***CI*** | ***p*** |  | ***M_Diff_*** | ***CI*** | ***p*** |  | ***M_Diff_*** | ***CI*** | ***p*** |
| Season ^a^ | | | |  |  |  |  |  |  |  |  |  |  |  |  |  |  |
|  | Spring | Winter |  |  |  |  |  |  |  |  | **-.27 ^c^** | [-.50, -.04] | .014 |  |  |  |  |
|  |  | Summer |  |  |  |  |  |  |  |  | **-.27 ^c^** | [-.47, -.06] | .005 |  | **-.30 ^d^** | [-.51, -.08] | .003 |
|  |  | Fall |  |  |  |  |  |  |  |  | **-.24 ^c^** | [-.47, -.01] | .036 |  | **-.36 ^d^** | [-.56, -.16] | < .001 |
| Birth mode ^b^ | | |  |  |  |  |  |  |  |  |  |  |  |  |  |  |  |
|  | Planned  C- section | Spontaneous  vaginal birth | **-157.83 ^d^** | [-258.18, -57.47] | .002 |  |  |  |  |  |  |  |  |  |  |  |  |
|  |  | Instrumental  vaginal birth | **-321.58 ^d^** | [-595.16, -48.00] | .020 |  | **-.37 ^c^** | [-.62, -.13] | < .001 |  |  |  |  |  |  |  |  |
|  | Instrument.vaginal birth | Spontaneous  vaginal birth |  |  |  |  | **.20 ^c^** | [.01, .38] | .027 |  |  |  |  |  |  |  |  |

*Note*. T1 DREAM_HAIR-BABY_ (*M* = 10.22 days after birth, *SD* = 4.11, *Range* = 0–21). T2 DREAM_HAIR-BABY_ (*M* = 8.40 weeks after birth, *SD* = 1.24, *Range* = 7–14). T3 DREAM_HAIR-BABY_ (*M* = 13.89 months after birth, *SD* = 0.62, *Range* = 12–16). T4 DREAM_HAIR-BABY_ (*M* = 23.88 months after birth, *SD* = 0.59, *Range* = 23–26). HairF = hair cortisol. Values in brackets show the 95% confidence interval for each analysis.

^a^ Winter is the reference category.

^b^ Spontaneous vaginal birth is the reference category.

^c^ Šidák- corrected post-hoc analysis.

^d^ Games-Howell- corrected post-hoc analysis.

^1^ *n* = 210, *n* varied slightly due to missing data. ^2^ *n* = 240, *n* varied slightly due to missing data. ^3^ *n* = 245. ^4^ *n* = 219.

**Appendix F. Results of *t*-Tests, ANOVAs and post-hoc tests for HairE**

**Table F.1 *t*-Test results for HairE and potential determinants in the postnatal period (T1**–**T4 DREAM_HAIR-BABY_)**

| HairE | | | | | | | | | | | | | | | | |
| --- | --- | --- | --- | --- | --- | --- | --- | --- | --- | --- | --- | --- | --- | --- | --- | --- |
|  |  | T1^1^ | | |  | T2^2^ | | |  | T3^3^ | | |  | T4^4^ | | |
| **Variable** |  | ***M(SD)*** | ***t df)*** | ***p*** |  | ***M(SD)*** | ***t (df)*** | ***p*** |  | ***M(SD)*** | ***t (df)*** | ***p*** |  | ***M(SD)*** | ***t (df)*** | ***p*** |
| Sex |  |  | -1.87(195) | .063 |  |  | -.67  (228) | .502 |  |  | .88(237) | .379 |  |  | .27(211) | .791 |
|  | Female | -.03 (.20) |  |  |  | -.01 (.34) |  |  |  | .01 (.36) |  |  |  | 1.52 (43.98) |  |  |
|  | Male | .03 (.24) |  |  |  | .02 (.28) |  |  |  | -.03 (.39) |  |  |  | -.09 (44.08) |  |  |
| Natural hair color | |  | -.52(186) | .602 |  |  | 1.30  (211) | .196 |  |  | .30(222) | .767 |  |  | -1.17(205) | .244 |
|  | Light | -.02 (.24) |  |  |  | .03 (.33) |  |  |  | .01 (.36) |  |  |  | -1.38 (43.12) |  |  |
|  | Dark | .00 (.22) |  |  |  | -.03 (.33) |  |  |  | -.01 (.41) |  |  |  | 7.61 (46.30) |  |  |
| COVID-19 pandemic exposure | | | -.17(207) | .867 |  |  | -.18  (237) | .855 |  |  | .14(243) | .885 |  |  | .19(217) | .853 |
|  | No | .00 (.24) |  |  |  | .00 (.31) |  |  |  | .01 (.39) |  |  |  | 3.63 (64.42) |  |  |
|  | Yes | .00 (.21) |  |  |  | .01 (.34) |  |  |  | .00 (.37) |  |  |  | -.08 (43.63) |  |  |
| *Note*. T1 DREAM_HAIR-BABY_ (*M* = 10.22 days after birth, *SD* = 4.11, *Range* = 0–21). T2 DREAM_HAIR-BABY_ (*M* = 8.40 weeks after birth, *SD* = 1.24, *Range* = 7–14). T3 DREAM_HAIR-BABY_ (*M* = 13.89 months after birth, *SD* = 0.62, *Range* = 12–16). T4 DREAM_HAIR-BABY_ (*M* = 23.88 months after birth, *SD* = 0.59, *Range* = 23–26). HairE = hair cortisone.  ^1^ *n* = 210, *n* varied slightly due to missing data. ^2^ *n* = 240, *n* varied slightly due to missing data. ^3^ *n* = 245. ^4^ *n* = 219. | | | | | | | | | | | | | | | | |

**Table F.2 ANOVA results for the influence of potential determinants on HairE during the postnatal period (T1**–**T4 DREAM_HAIR-BABY_)**

| HairE | | | | | | | | | | | | | | | | | | | |
| --- | --- | --- | --- | --- | --- | --- | --- | --- | --- | --- | --- | --- | --- | --- | --- | --- | --- | --- | --- |
| **Variable** | T1^1^ | | | |  | T2^2^ | | | |  | T3^3^ | | | |  | T4^4^ | | | |
|  | ***F*** | ***df ^a^*** | ***df ^b^*** | ***Sig*** |  | ***F*** | ***df ^a^*** | ***df ^b^*** | ***Sig*** |  | ***F*** | ***df ^a^*** | ***df ^b^*** | ***Sig*** |  | ***F*** | ***df ^a^*** | ***df ^b^*** | ***Sig*** |
| Season ^c^ | 2.40 ^e^ | 3 | 113.21 | .071 |  | 2.27 | 3 | 235 | .081 |  | **5.68** | 3 | 241 | <.001 |  | **8.80** ^e^ | 3 | 117.05 | <.001 |
| Birth mode ^d^ | .72 | 3 | 193 | .539 |  | .10 | 3 | 224 | .958 |  | .82 | 3 | 235 | .482 |  | 1.18 | 3 | 208 | .320 |

*Note*. T1 DREAM_HAIR-BABY_ (*M* = 10.22 days after birth, *SD* = 4.11, *Range* = 0–21). T2 DREAM_HAIR-BABY_ (*M* = 8.40 weeks after birth, *SD* = 1.24, *Range*
= 7–14). T3 DREAM_HAIR-BABY_ (*M* = 13.89 months after birth, *SD* = 0.62, *Range* = 12–16). T4 DREAM_HAIR-BABY_ (*M* = 23.88 months after birth, *SD* = 0.59, *Range* = 23–26). HairE = hair cortisone. Significant associations presented in bold.

^a^ Df between the groups.

^b^ Df within the groups.

^c^ includes: Winter (reference category), Spring, Summer, Fall.

^d^ includes: Spontaneous vaginal birth (reference category), Instrumental vaginal birth, Planned cesarean section, Unplanned cesarean section.

^e^ WELCH-ANOVA is reported because of inhomogenity of variance.

^1^ *n* = 210, *n* varied slightly due to missing data. ^2^ *n* = 240, *n* varied slightly due to missing data. ^3^ *n* = 245. ^4^ *n* = 219.

**Table F.3 Post-hoc analysis for HairE and potential determinants in the postnatal period (T3**–**T4 DREAM_HAIR-BABY_)**

| HairE | | | | | | | | | |
| --- | --- | --- | --- | --- | --- | --- | --- | --- | --- |
|  | |  | T3^1^ | | |  | T4^2^ | | |
| **Comparison** | |  | ***M_Diff_*** | ***CI*** | ***p*** |  | ***M_Diff_*** | ***CI*** | ***p*** |
| Season ^a^ | | |  |  |  |  |  |  |  |
|  | Spring | Winter | **-.20 ^b^** | [-.38, -.02] | .021 |  |  |  |  |
|  |  | Summer | **-.23 ^b^** | [-.40, -.07] | .001 |  | **-28.35 ^c^** | [-47.18, -9.52] | < .001 |
|  |  | Fall | **-.21 ^b^** | [-.40, -.03] | .012 |  | **-34.14 ^c^** | [-54.86, -13.42] | < .001 |
|  | Fall | Winter |  |  |  |  | **26.72 ^c^** | [3.82, 49.61] | .015 |

*Note*. T3 DREAM_HAIR-BABY_ (*M* = 13.89 months after birth, *SD* = 0.62, *Range* = 12–16). T4 DREAM_HAIR-BABY_ (*M* = 23.88

months after birth, *SD* = 0.59, *Range* = 23–26). HairE = hair cortisone. Values in brackets show the 95%

confidence interval for each analysis.

^a^ Winter is the reference category.

^b^ Šidák- corrected post-hoc analysis.

^c^ Games-Howell- corrected post-hoc analysis.

^1^ *n* = 245. ^2^ *n* = 219.

**Appendix G. Results of *t*-Tests, ANOVAs and post-hoc tests for HairDHEA**

**Table G.1 *t*-Test results for HairDHEA and potential determinants in the postnatal period (T1**–**T4 DREAM_HAIR-BABY_)**

| HairDHEA | | | | | | | | | | | | | | | | | |
| --- | --- | --- | --- | --- | --- | --- | --- | --- | --- | --- | --- | --- | --- | --- | --- | --- | --- |
|  | |  | T1^1^ | | |  | T2^2^ | | |  | T3^3^ | | |  | T4^4^ | | |
| **Variable** | |  | ***M(SD)*** | ***t df)*** | ***p*** |  | ***M(SD)*** | ***t (df)*** | ***p*** |  | ***M(SD)*** | ***t (df)*** | ***p*** |  | ***M(SD)*** | ***t (df)*** | ***p*** |
| Sex |  | |  | .68(195) | .500 |  |  | .19(228) | .848 |  |  | -1.78  (237) | .076 |  |  | **-2.64**(211) | .009 |
|  | Female | | .01 (.25) |  |  |  | .01 (.37) |  |  |  | -.08 (.66) |  |  |  | -.09 (.52) |  |  |
|  | Male | | -.02 (.24) |  |  |  | .00 (.38) |  |  |  | .08 (.67) |  |  |  | .10 (.53) |  |  |
| Natural hair color | | |  | -1.06(186) | .290 |  |  | -1.31  (211) | .191 |  |  | .18(222) | .861 |  |  | .26(205) | .795 |
|  | Light | | -.02 (.23) |  |  |  | -.03 (.36) |  |  |  | .01 (.67) |  |  |  | .01 (.53) |  |  |
|  | Dark | | .02 (.25) |  |  |  | .03 (.37) |  |  |  | -.01 (.70) |  |  |  | -.01 (.53) |  |  |
| COVID-19 pandemic exposure | | | | .36(207) | .718 |  |  | -.26  (237) | .794 |  |  | -.21  (243) | .837 |  |  | -1.16(217) | .248 |
|  | No | | .00 (.25) |  |  |  | .00 (.38) |  |  |  | -.01 (.45) |  |  |  | -.27 (.13) |  |  |
|  | Yes | | -.01 (.26) |  |  |  | .01 (.34) |  |  |  | .01 (.74) |  |  |  | .01 (.53) |  |  |
| *Note*. T1 DREAM_HAIR-BABY_ (*M* = 10.22 days after birth, *SD* = 4.11, *Range* = 0–21). T2 DREAM_HAIR-BABY_ (*M* = 8.40 weeks after birth, *SD* = 1.24, *Range* = 7–14). T3 DREAM_HAIR-BABY_ (*M* = 13.89 months after birth, *SD* = 0.62, *Range* = 12–16). T4 DREAM_HAIR-BABY_ (*M* = 23.88 months after birth, *SD* = 0.59, *Range* = 23–26). HairDHEA = hair dehydroepiandrosterone. Significant associations presented in bold.  ^1^ *n* = 210, *n* varied slightly due to missing data. ^2^ *n* = 240, *n* varied slightly due to missing data. ^3^ *n* = 245. ^4^ *n* = 219. | | | | | | | | | | | | | | | | | |

**Table G.2 ANOVA results for the influence of potential determinants on HairDHEA during the postnatal period (T1**–**T4 DREAM_HAIR-BABY_)**

| HairDHEA | | | | | | | | | | | | | | | | | | | |
| --- | --- | --- | --- | --- | --- | --- | --- | --- | --- | --- | --- | --- | --- | --- | --- | --- | --- | --- | --- |
| **Variable** | T1^1^ | | | |  | T2^2^ | | | |  | T3^3^ | | | |  | T4^4^ | | | |
|  | ***F*** | ***df ^a^*** | ***df ^b^*** | ***Sig*** |  | ***F*** | ***df ^a^*** | ***df ^b^*** | ***Sig*** |  | ***F*** | ***df ^a^*** | ***df ^b^*** | ***Sig*** |  | ***F*** | ***df ^a^*** | ***df ^b^*** | ***Sig*** |
| Season ^c^ | 1.95 | 3 | 205 | .123 |  | .69 | 3 | 235 | .558 |  | **9.72** | 3 | 241 | <.001 |  | **8.99** | 3 | 215 | <.001 |
| Birth mode ^d^ | .96 | 3 | 193 | .415 |  | 1.72 | 3 | 224 | .164 |  | 1.47 | 3 | 235 | .224 |  | .80 | 3 | 208 | .497 |

*Note*. T1 DREAM_HAIR-BABY_ (*M* = 10.22 days after birth, *SD* = 4.11, *Range* = 0–21). T2 DREAM_HAIR-BABY_ (*M* = 8.40 weeks after birth, *SD* = 1.24, *Range*
= 7–14). T3 DREAM_HAIR-BABY_ (*M* = 13.89 months after birth, *SD* = 0.62, *Range* = 12–16). T4 DREAM_HAIR-BABY_ (*M* = 23.88 months after birth, *SD* = 0.59, *Range* = 23–26). HairDHEA = hair dehydroepiandrosterone. Significant associations presented in bold.

^a^ Df between the groups.

^b^ Df within the groups.

^c^ includes: Winter (reference category), Spring, Summer, Fall.

^d^ includes: Spontaneous vaginal birth (reference category), Instrumental vaginal birth, Planned cesarean section, Unplanned cesarean section.

^1^ *n* = 210, *n* varied slightly due to missing data. ^2^ *n* = 240, *n* varied slightly due to missing data. ^3^ *n* = 245. ^4^ *n* = 219.

**Table G.3 Post-hoc analysis for HairDHEA and potential determinants in the postnatal period (T3**–**T4 DREAM_HAIR-BABY_)**

| HairDHEA | | | | | | | | | |
| --- | --- | --- | --- | --- | --- | --- | --- | --- | --- |
|  | |  | T3^1^ | | |  | T4^2^ | | |
| **Comparison** | |  | ***M_Diff_*** | ***CI*** | ***p*** |  | ***M_Diff_*** | ***CI*** | ***p*** |
| Season ^a^ | | |  |  |  |  |  |  |  |
|  | Summer | Winter | **.30 ^b^** | [.00, .60] | .049 |  | **.40 ^b^** | [.14, .66] | < .001 |
|  |  | Spring | **.53 ^b^** | [.24, .81] | < .001 |  | **.30 ^b^** | [.06, .55] | .008 |
|  | Fall | Winter |  |  |  |  | **.40 ^b^** | [.13, .66] | < .001 |
|  |  | Spring | **.49 ^b^** | [.17, .80] | < .001 |  | **.30 ^b^** | [.05, .54] | .010 |

Note. T3 DREAM_HAIR-BABY_ (M = 13.89 months after birth, SD = 0.62, Range = 12–16). T4 DREAM_HAIR-BABY_ (M = 23.88 months

after birth, SD = 0.59, Range = 23–26). HairDHEA = hair dehydroepiandrosterone. Values in brackets show the 95% confidence

interval for each analysis.

^a^ Winter is the reference category.

^b^ Šidák- corrected post-hoc analysis.

^1^ *n* = 245. ^2^ *n* = 219.

**Appendix H. Results of *t*-Tests, ANOVAs and post-hoc tests for HairP**

**Table H.1 *t*-Test results for HairP and potential determinants in the postnatal period (T1**–**T4 DREAM_HAIR-BABY_)**

| HairP | | | | | | | | | | | | | | | | |
| --- | --- | --- | --- | --- | --- | --- | --- | --- | --- | --- | --- | --- | --- | --- | --- | --- |
|  |  | T1^1^ | | |  | T2^2^ | | |  | T3^3^ | | |  | T4^4^ | | |
| **Variable** |  | ***M(SD)*** | ***t df)*** | ***p*** |  | ***M(SD)*** | ***t (df)*** | ***p*** |  | ***M(SD)*** | ***t (df)*** | ***p*** |  | ***M(SD)*** | ***t (df)*** | ***p*** |
| Sex |  |  | **2.12(**195) | .035 |  |  | **2.72**  (228) | .007 |  |  | -.98  (237) | .328 |  |  | -.98(211) | .329 |
|  | Female | 48.52 (314.75) |  |  |  | .10 (.59) |  |  |  | -.05 (.74) |  |  |  | -.06 (.81) |  |  |
|  | Male | -49.58 (331.78) |  |  |  | -.11 (.61) |  |  |  | .05 (.88) |  |  |  | .05 (.82) |  |  |
| Natural hair color | | | **-3.09**(186) | .002 |  |  | **-2.68**  (211) | .008 |  |  | **2.26**  (222) | .025 |  |  | .21(205) | .833 |
|  | Light | -95.66 (305.07) |  |  |  | -.13 (.59) |  |  |  | .05 (.74) |  |  |  | .00 (.80) |  |  |
|  | Dark | 57.75 (327.87) |  |  |  | .09 (.61) |  |  |  | -.25 (.95) |  |  |  | -.03 (.83) |  |  |
| COVID-19 pandemic exposure | | | -.74(207) | .459 |  |  | .15(237) | .881 |  |  | .03(243) | .976 |  |  | .24(217) | .808 |
|  | No | -9.54 (338.07) |  |  |  | .00 (.62) |  |  |  | .00 (.54) |  |  |  | .09 (.62) |  |  |
|  | Yes | 30.34 (308.85) |  |  |  | -.01 (.61) |  |  |  | .00 (.88) |  |  |  | .00 (.82) |  |  |
| *Note*. T1 DREAM_HAIR-BABY_ (*M* = 10.22 days after birth, *SD* = 4.11, *Range* = 0–21). T2 DREAM_HAIR-BABY_ (*M* = 8.40 weeks after birth, *SD* = 1.24, *Range* = 7–14).  T3 DREAM_HAIR-BABY_ (*M* = 13.89 months after birth, *SD* = 0.62, *Range* = 12–16). T4 DREAM_HAIR-BABY_ (*M* = 23.88 months after birth, *SD* = 0.59, *Range* = 23–26).  HairP = hair progesterone. Significant associations presented in bold.  ^1^ *n* = 210, *n* varied slightly due to missing data. ^2^ *n* = 240, *n* varied slightly due to missing data. ^3^ *n* = 245. ^4^ *n* = 219. | | | | | | | | | | | | | | | | |

**Table H.2 ANOVA results for the influence of potential determinants on HairP during the postnatal period (T1**–**T4 DREAM_HAIR-BABY_)**

| HairP | | | | | | | | | | | | | | | | | | | |
| --- | --- | --- | --- | --- | --- | --- | --- | --- | --- | --- | --- | --- | --- | --- | --- | --- | --- | --- | --- |
| **Variable** | T1^1^ | | | |  | T2^2^ | | | |  | T3^3^ | | | |  | T4^4^ | | | |
|  | ***F*** | ***df ^a^*** | ***df ^b^*** | ***Sig*** |  | ***F*** | ***df ^a^*** | ***df ^b^*** | ***Sig*** |  | ***F*** | ***df ^a^*** | ***df ^b^*** | ***Sig*** |  | ***F*** | ***df ^a^*** | ***df ^b^*** | ***Sig*** |
| Season ^c^ | **3.22** | 3 | 205 | .024 |  | **3.34** ^e^ | 3 | 122.30 | .022 |  | 1.92 ^e^ | 3 | 122.67 | .130 |  | 1.44 | 3 | 215 | .231 |
| Birth mode ^d^ | 2.38 | 3 | 193 | **.**071 |  | 0.75 | 3 | 224 | .523 |  | .62 | 3 | 235 | .603 |  | .41 | 3 | 208 | .750 |

*Note*. T1 DREAM_HAIR-BABY_ (*M* = 10.22 days after birth, *SD* = 4.11, *Range* = 0–21). T2 DREAM_HAIR-BABY_ (*M* = 8.40 weeks after birth, *SD* = 1.24, *Range* = 7–14). T3 DREAM_HAIR-BABY_ (*M* = 13.89 months after birth, *SD* = 0.62, *Range* = 12–16). T4 DREAM_HAIR-BABY_ (*M* = 23.88 months after birth, *SD* = 0.59,

*Range* = 23–26). HairP = hair progesterone. Significant associations presented in bold.

^a^ Df between the groups.

^b^ Df within the groups.

^c^ includes: Winter (reference category), Spring, Summer, Fall.

^d^ includes: Spontaneous vaginal birth (reference category), Instrumental vaginal birth, Planned cesarean section, Unplanned cesarean section.

^e^ WELCH-ANOVA is reported because of inhomogenity of variance.

^1^ *n* = 210, *n* varied slightly due to missing data. ^2^ *n* = 240, *n* varied slightly due to missing data. ^3^ *n* = 245. ^4^ *n* = 219.

**Table H.3 Post-hoc analysis for HairP and potential determinants in the postnatal period (T1**–**T2 DREAM_HAIR-BABY_)**

| HairP | | | | | | | | | |
| --- | --- | --- | --- | --- | --- | --- | --- | --- | --- |
|  | |  | T1^1^ | | |  | T2^2^ | | |
| **Comparison** | |  | ***M_Diff_*** | ***CI*** | ***p*** |  | ***M_Diff_*** | ***CI*** | ***p*** |
| Season ^a^ | | |  |  |  |  |  |  |  |
|  | Spring | Summer | **174.56 ^b^** | [14.35, 334.78] | .025 |  | **.26 ^c^** | [.00, .52] | .050 |

*Note*. T1 DREAM_HAIR-BABY_ (*M* = 10.22 days after birth, *SD* = 4.11, *Range* = 0–21). T2 DREAM_HAIR-BABY_ (*M* = 8.40 weeks

after birth, *SD* = 1.24, *Range* = 7–14). HairP = hair progesterone. Values in brackets show the 95% confidence interval for

each analysis.

^a^ Winter is the reference category.

^b^ Šidák- corrected post-hoc analysis.

^c^ Games-Howell- corrected post-hoc analysis.

^1^ *n* = 210, *n* varied slightly due to missing data. ^2^ *n* = 240, *n* varied slightly due to missing data.

**Appendix I. Results from exploratory analyses regarding APGAR score and hair steroid concentrations**

**Table I.1** Spearman rank correlations [*95% BCa CI ^a^*] between the APGAR score and hair steroids (T1–T4 DREAM_HAIR-BABY_)

|  | **T1*^1^*** | | | | **T2*^2^*** | | | **T3*^3^*** | | | | **T4*^4^*** | | | |
| --- | --- | --- | --- | --- | --- | --- | --- | --- | --- | --- | --- | --- | --- | --- | --- |
| **Hair steroid** | ***r*** | ***p*** | ***CI*** |  | ***r*** | ***p*** | ***CI*** |  | ***r*** | ***p*** | ***CI*** |  | ***r*** | ***p*** | ***CI*** |
| HairF | -.02 | .469 | [-.17, .12] |  | -.08 | .294 | [-.21, .06] |  | -.06 | .377 | [-.19, .07] |  | -.07 | .354 | [-.21, .06] |
| HairE | **-.21** | **.038** | [-.35, -.06] |  | -.05 | .419 | [-.20, .07] |  | -.07 | .363 | [-.19, .07] |  | -.07 | .358 | [-.22, .06] |
| HairDHEA | -.07 | .356 | [-.23, .08] |  | -.05 | .431 | [-.17, .10] |  | -.05 | .430 | [-.18, .07] |  | -.09 | .324 | [-.24, .05] |
| HairP | .08 | .344 | [-.08, .23] |  | .01 | .479 | [-.14, .14] |  | .03 | .453 | [-.11, .15] |  | .02 | .489 | [-.12, .16] |

*Note*. T1 DREAM_HAIR-BABY_ (*M* = 10.22 days after birth, *SD* = 4.11, *Range* = 0–21). T2 DREAM_HAIR-BABY_ (*M* = 8.40 weeks after birth, *SD* = 1.24, *Range* = 7–14).
T3 DREAM_HAIR-BABY_ (*M* = 13.89 months after birth, *SD* = 0.62, *Range* = 12–16). T4 DREAM_HAIR-BABY_ (*M* = 23.88 months after birth, *SD* = 0.59, *Range* = 23–26).
HairF = hair cortisol; HairE = hair cortisone; HairDHEA = hair dehydroepiandrosterone; HairP = hair progesterone. Significant associations (*p <* .05, two-tailed) presented

in bold.

^a^ Values in brackets show the 95% confidence interval for each correlation (bias-corrected and accelerated bootstrap method; 2000 iterations).

^1^ *n* = 174-209. ^2^ *n* = 205-240. ^3^ *n* = 213-245. ^4^ *n* = 178-219. *n* varied due to missing data within each time point.

**Appendix J. Summary of significant findings from the regression analyses**

**Table J.1** Factors associated with hair steroid levels: developmental factors as well as methodological and contextual factors

|  |  | Hair steroid  Variable | HairF | HairE | HairDHEA | HairP |
| --- | --- | --- | --- | --- | --- | --- |
| Developmental factors | Sociodemographic determinants | Age |  |  |  |  |
|  |  | Sex^a^ |  |  | T4: *β* = .15, *p* = .023^e^ |  |
|  | Birth-related determinants | Weight |  |  |  |  |
|  |  | Gestational week | T1: *β* = .24, *p* = .003^e^ |  |  |  |
|  |  | Birth mode^b^ | T1: *β* = -.13, *p* = <.001  T2: *β* = .17, *p* = .009^e^  *β* = -.13, *p* = .016^e^ |  |  |  |
|  |  | APGAR score |  |  |  |  |
| Methodological and Contextual factors | Hair-related determinants | Hair-washing frequency |  |  |  |  |
|  |  | Natural hair color^c^ |  |  |  | T1: *β* = .20, *p* = .012^e^  T2: *β* = .18, *p* = .008^e^ |
|  |  | Sunlight exposure |  |  |  |  |
|  |  | Season^d^ |  | T4: *β* = .19, *p* = .022^e^ | T3: *β* = .21, *p* = .012^e^  *β* = .16, *p* = .04^e^  T4: *β* = .26, *p* = .002  *β* = .21, *p* = .012^e^ |  |
|  | Organisational determinants | Storage time |  |  | T4: *β* = -.16, *p* = .038^e^ |  |
|  | Covid-19 pandemic | COVID-19 pandemic |  |  |  |  |

*Note.* T1 DREAM_HAIR-BABY_ (*M* = 10.22 days after birth, *SD* = 4.11, *Range* = 0–21). T2 DREAM_HAIR-BABY_ (*M* = 8.40 weeks after birth, *SD* = 1.24, *Range* = 7–14). T3 DREAM_HAIR-BABY_

(*M* = 13.89 months after birth, *SD* = 0.62, *Range* = 12–16). T4 DREAM_HAIR-BABY_ (*M* = 23.88 months after birth, *SD* = 0.59, *Range* = 23–26). *β* = Standardised beta coefficient.

HairF = hair cortisol; HairE = hair cortisone; HairDHEA = hair dehydroepiandrosterone; HairP = hair progesterone.

^a^ Reference category is the female sex.

^b^ Reference category is unassisted vaginal birth. Other categories include instrumental vaginal birth, planned C-section, unplanned C-section. At T1 and T2, Planned C-section presented lower HairF compared to unassisted or instrumental vaginal birth. At T2 instrumental vaginal birth showed higher HairF compared to unassisted vaginal birth or planned C-section.

^c^ Reference category is light hair color (blond, dark blond, red-blond).

^d^ Reference category is winter. Other categories include spring, summer, and fall. At T3 and T4, HairDHEA levels were higher in both summer and fall compared to winter.

^e^ Did not survive Šidák correction.

**References**

[1] Corona-Zahlen: Montag, 16.1.2023 – Das war‘s (erstmal) - WELT. DIE WELT 2026. https://www.welt.de/wirtschaft/article238812729/Corona-Zahlen-Montag-16-1-2023-Das-war-s-erstmal.html (accessed March 4, 2026).

[2] Khailaie S, Mitra T, Bandyopadhyay A, Schips M, Mascheroni P, Vanella P, et al. Development of the reproduction number from coronavirus SARS-CoV-2 case data in Germany and implications for political measures. BMC Med 2021;19:32. https://doi.org/10.1186/s12916-020-01884-4.
